# Cell reprogramming: methods, mechanisms and applications

**DOI:** 10.1186/s13619-025-00229-x

**Published:** 2025-03-27

**Authors:** Fei Zhu, Guangjun Nie

**Affiliations:** 1 Wisdom Lake Academy of Pharmacy, Xi’an Jiaotong-Liverpool University, Suzhou, 215123 China; 2 CAS Key Laboratory for Biomedical Effects of Nanomaterials and Nanosafety, CAS Center of Excellence in Nanoscience National Center for Nanoscience and Technology, Beijing, 100190 China; 3 Center of Materials Science and Optoelectronics Engineering, University of Chinese Academy of Sciences, Beijing, 100049 China

**Keywords:** Cell reprogramming, Biophysical regulation, Disease modeling, Drug development, Cell therapy, Cancer immunotherapy

## Abstract

Cell reprogramming represents a powerful approach to achieve the conversion cells of one type into cells of another type of interest, which has substantially changed the landscape in the field of developmental biology, regenerative medicine, disease modeling, drug discovery and cancer immunotherapy. Cell reprogramming is a complex and ordered process that involves the coordination of transcriptional, epigenetic, translational and metabolic changes. Over the past two decades, a range of questions regarding the facilitators/barriers, the trajectories, and the mechanisms of cell reprogramming have been extensively investigated. This review summarizes the recent advances in cell reprogramming mediated by transcription factors or chemical molecules, followed by elaborating on the important roles of biophysical cues in cell reprogramming. Additionally, this review will detail our current understanding of the mechanisms that govern cell reprogramming, including the involvement of the recently discovered biomolecular condensates. Finally, the review discusses the broad applications and future directions of cell reprogramming in developmental biology, disease modeling, drug development, regenerative/rejuvenation therapy, and cancer immunotherapy.

## Background

How cell identity is decided and regulated is one of the fundamental questions in modern biology. During developmental progression, a continuum of cell fates is generated from the totipotent fertilized egg to terminally differentiated cells. It has been historically recognized that the stability of cell identity within a specialized tissue/organ is essential for maintaining structural and functional integrity (Alvarado and Yamanaka 2014). This recognition has not been changed until the influential concepts made by longstanding efforts of biologists that somatic cells in certain lower animal species have the ability to change their cellular identity for regeneration of injured tissues (Jopling et al. 2011). This ability of adaptive somatic cell reprogramming in higher mammals, is largely diminished (Wang et al. 2023b). The pioneering research on somatic cell nuclear transfer (SCNT) in amphibians and mammals demonstrated that undefined cell-intrinsic factors within oocyte enable efficient cell fate manipulation and cloning (Gurdon 1962; Campbell et al. 1996), which provides the fundamental principles for cell reprogramming.

The notion that transcription factors can change cell fate came from a preliminary study, in which the overexpression of myoblast-signature gene-encoded transcription factor MyoD converted murine embryonic fibroblasts into myoblasts (Davis et al. 1987). In a groundbreaking study, Yamanaka and colleagues reported the generation of induced pluripotent stem cells (iPSCs) from mouse somatic cells via forced expression of OCT4, SOX2, KLF4 and c-MYC (OSKM) (Takahashi and Yamanaka 2006). The following studies have reported the reprogramming of human somatic cells into iPSCs (Takahashi et al. 2007; Yu et al. 2007; Park et al. 2008b; Meissner et al. 2007), which opened avenues in disease modeling, drug discovery, and cell therapies. However, initial studies of cell reprogramming overexpressed OSKM delivered by retroviral or lentiviral vector, which has the genome-integrating risk of exogenous genes, especially *c-Myc*. Alternative approaches, such as non-integrative delivery tools, mRNA, and protein, have been developed to tackle the limitations as much as possible (González et al. 2011). The success in the reprogramming of mouse/human somatic cells into induced pluripotent stem cells by chemical cocktails offers an attractive approach to modulate cell fate (Guan et al. 2022; Hou et al. 2013; Liu et al. 2022). Unlike pluripotent reprogramming (namely reprogramming of somatic cells towards iPSCs), direct reprogramming (also known as transdifferentiation) is the process of cell fate conversion from cells of one lineage into cells of another with lineage-specific transcription factors or chemical cocktails bypassing an intermediate pluripotent or multipotent stage (Wang et al. 2021b). Numerous studies have reported the direct reprogramming of somatic cells towards neurons (Vierbuchen et al. 2010; Li et al. 2015; Jorstad et al. 2017; Pang et al. 2011), cardiomyocytes (Qian et al. 2012; Cao et al. 2016; Ieda et al. 2010), hepatocytes (Huang et al. 2011, 2014; Sekiya and Suzuki 2011; Xie et al. 2019), Sertoli cells (Liang et al. 2019), osteoblasts (Yamamoto et al. 2015), dendritic cells (Rosa et al. 2018), or natural killer cells (Kim et al. 2021) using combinatorial transcription factors or chemical cocktails (Table 1). This review summarizes the current advances in the use of cell reprogramming to generate cells of interest. The substantial progress in the field of cell reprogramming has offered versatile toolbox for developmental biology, disease modeling, drug discovery, regenerative medicine, cell therapy and tumor immunotherapy.

Besides biochemical cues, biophysical cues (such as substrate stiffness, topological cues, or microporosity) can be important driving forces in cell fate decisions, embryonic development, adult tissue homeostasis and disease progression (Keung et al. 2010; Zhu et al. 2023b; Song et al. 2020b). Growing evidence has built the insight that biophysical cues play fundamental roles in cell reprogramming (Downing et al. 2013; Wang et al. 2020b; Song et al. 2022; Qu et al. 2023; Zhu et al. 2024), highlighting the importance of optimizing both biophysical cues and biochemical signals to achieve optimal outcomes of cell reprogramming.

In this review, the author will describe the historical development of the field of cell reprogramming, highlighting the key discoveries. The author then will detail different approaches to cell reprogramming and elaborate on the molecular and cellular mechanisms governing cell reprogramming. Finally, the widespread applications and future directions of cell reprogramming in developmental biology, disease modeling, drug development, regenerative medicine, cell therapy and cancer immunotherapy will be discussed.

## Methods for cell reprogramming

### Methods for pluripotent reprogramming

#### Transcription factor-mediated pluripotent reprogramming

**Fig. 1:**
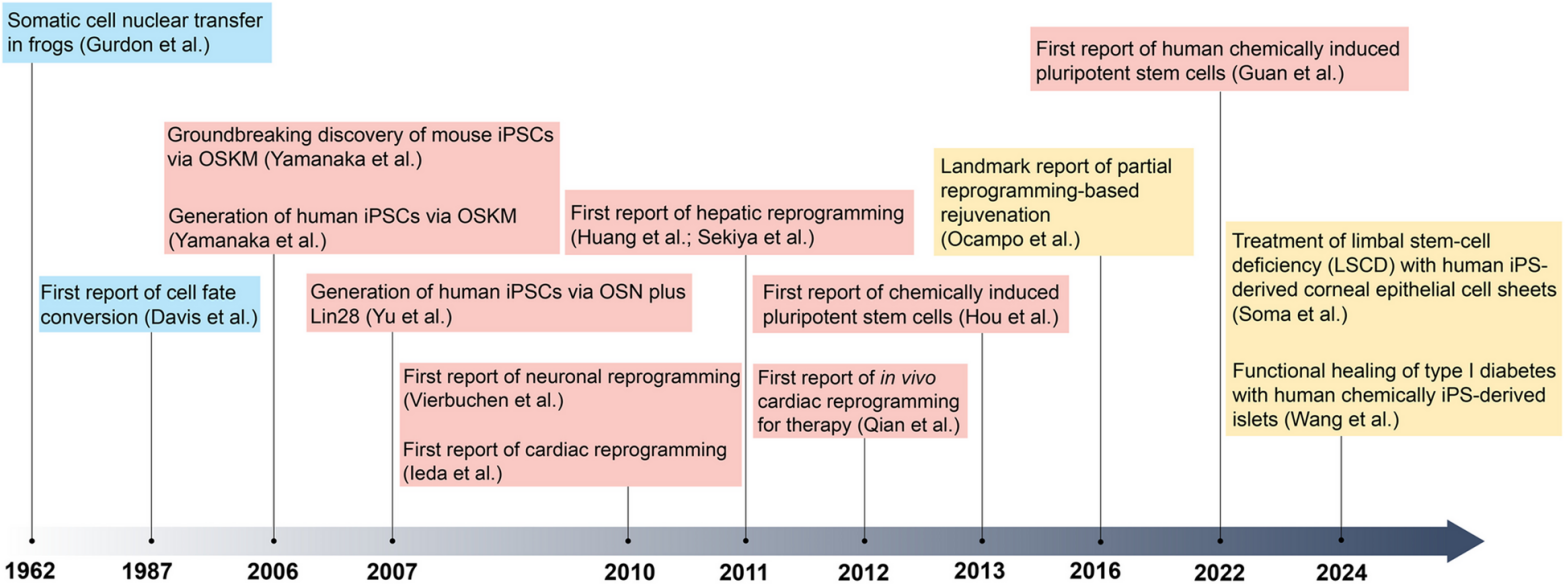
Timeline of key advances in cell reprogramming or cell reprogramming-based therapies. The diagram shows the achievements and milestones in research on cell reprogramming. OKSM: Oct4, Klf4, Sox2, c-Myc. OSN: Oct4, Sox2, Nanog

Conrad Waddington proposed a model that depicted the irreversible pattern of terminally differentiated cells during embryonic development as a ball rolling downhill towards a more and more restricted state (Waddington 2014). By performing somatic cell nuclear transfer (SCNT) experiments using *Xenopus laevis* frog in 1962, John Gurdon demonstrated that the nucleus isolated from terminally differentiated somatic cell harbored all the genetic information required for successful cloning and giving rise to sexually mature organisms (Fig. 1), suggesting the reversible pattern of the mechanisms governing somatic cell fate (Gurdon 1962). This notion was reinforced by somatic cell nuclear transfer (SCNT) in sheep by Wilmut and colleagues (Campbell et al. 1996). The insight that transcription factors can modulate cell fate came from a seminal study (Fig. 1), in which the stable conversion of murine embryonic fibroblasts into myoblasts was achieved via ectopic expression of myoblast signature gene-encoded transcription factor (MyoD1) (Davis et al. 1987). Another elegant research successfully reprogrammed B cells into macrophages via overexpression of the C/EBPα/β transcription factors, further revealing the important role of transcription factors in modulating cell fate (Xie et al. 2004). In Yamanaka’s groundbreaking study, forced expression of OCT4, SOX2, KLF4 and c-MYC (OSKM) could directly reprogram mouse and human somatic cells towards induced pluripotent stem cells (iPSCs) (Fig. 1), which were highly similar to embryonic stem cells in molecular and functional level (Takahashi and Yamanaka 2006). iPSCs are morphologically and functionally similar to embryonic stem cells (ESCs) (Takahashi and Yamanaka 2006; Takahashi et al. 2007; Yu et al. 2007; Park et al. 2008b), and have been demonstrated to be truly pluripotent in tetraploid complementation assay (Zhao et al. 2009; Kang et al. 2009).

Since the first report of iPSCs by Yamanaka, alternative combinations of transcription factors have been developed (Table 1). For example, the induction of pluripotency could also be achieved by the combination of Oct4, Sox2 and Esrrb (Feng et al. 2009). Another study found that Nr5a2, could replace exogenous Oct4 and worked in conjunction with Sox2 and Klf4 to mediate the successful generation of iPSCs from murine embryonic fibroblasts (Heng et al. 2010). In a proof-of-principle study led by Jaenisch, the authors generated high-quality iPSCs with a completely different set of transcription factors: Sall4, Nanog, Esrrb and Lin28 (Buganim et al. 2014). The authors further demonstrated in tetraploid complementation assay that one major determinant of the quality of iPSCs was the combination of reprogramming factors used (Buganim et al. 2014). Notably, Montserrat and colleagues identified several factors that were traditionally related to lineage specification, which also allowed for the replacement of the core pluripotency factors SOX2 and of OCT4 in the reprogramming of human fibroblasts to iPSCs (Montserrat et al. 2013). Consistent with this observation, Shu and co-workers provided evidence that concurrent coexpression of *Gata3* and *Gmnn* could substitute for *Oct4* and *Sox2* as reprogramming factors for inducing mouse fibroblasts towards iPSCs (Shu et al. 2015). In a recent study, Wang and colleagues presented evidence that efficient reprogramming of somatic cells was driven by a novel combination of transcription factors: Jdp2, Glis1, Esrrb and Sall4 (Wang et al. 2023a). Mechanistic studies unveiled that Sall4 recruited Nucleosome Remodeling and histone Deacetylation (NuRD) complex to open chromatin in MEFs to ensure the closure of somatic loci during cell reprogramming (Wang et al. 2023a). Synthetic reprogramming factors have been demonstrated to induce pluripotency faster and more efficiently than time-consuming and inefficient cell reprogramming mediated by Yamanaka factors (OSKM). For example, Wang and colleagues described the robust and reproducible generation of iPSCs by fusing the transactivation domain of VP16 to OCT4, NANOG and SOX2, respectively (Wang et al. 2011b). In another notable study, Zhu and co-workers developed a novel combination of synthetic reprogramming factors (OySyNyK) via fusing the transactivation domain of the Yes-associated protein (YAP) to defined factors and established a highly efficient and rapid reprogramming system (Zhu et al. 2014). The authors demonstrated that the efficiency of OySyNyK-induced iPSCs was up to 100-fold higher than the OSNK-mediated reprogramming (Zhu et al. 2014). The activation of endogenous Oct4 was very rapid and initiated within 24 h post-infection of OySyNyK. Mechanistic studies revealed the coordination of engineered factors with TETs to regulate 5hmC-mediated epigenetic control underlying this highly efficient and rapid reprogramming system (Zhu et al. 2014). Taken together, these studies provide solid evidence that the process of pluripotency acquisition from somatic cells harbors a remarkable degree of flexibility in terms of the combinations of reprogramming transcription factors used.

**Table 1:** Summary of methods for improving the efficiency of cell reprogramming

Starting cell types	Reprogramming combination(s)	Ending cell types	Scoring criteria	Reprogramming efficiency	Pros and Cons	References
MEFs or TTFs	Oct4, Sox2, Klf4, c-Myc	iPSCs	Fbx15-positive colonies	~ 0.1%	**Cons**: Use of retrovirus; Time-consuming and low efficiency	Takahashi and Yamanaka 2006
HDFs	Oct4, Sox2, Klf4, c-Myc	iPSCs	ES-like colonies	~ 0.02%	**Cons**: Use of retrovirus; Time-consuming and low efficiency	Takahashi et al. 2007
IMR90, Human foreskin fibroblasts	Oct4, Sox2, Nanog, Lin28	iPSCs	Oct4-positive colonies	0.01%−0.022%	**Cons**: Use of lentivirus; Time-consuming and low efficiency	Yu et al. 2007
HFF or HDF	Oct4, Sox2 plus VPA	iPSCs	ES-like colonies	0.73%−1.1%	**Pros**: High efficiency **Cons**: Use of retrovirus	Huangfu et al. 2008a, b
MEFs, Human skin fibroblasts	Oct4, Sox2, Klf4, c-Myc plus Vc, VPA	iPSCs	Oct4-positive colonies (mouse); AP-positive colonies (human)	Mouse: 8.75% Human: 7.05%	**Pros**: High efficiency **Cons**: Use of retrovirus	Esteban et al. 2010
MEFs, HFFs	Oct4-VP16, Sox2-VP16, Klf4, Nanog-VP16	iPSCs	Oct4-positive colonies; Human ES-like colonies	Mouse: ~ 1.12% Human: ~ 0.24%	**Pros**: Fast, high efficiency and non-integration	Wang et al. 2011a, b
MEFs	Oct4-YAP-TAD, Sox2- YAP-TAD, Klf4, Nanog- YAP-TAD	iPSCs	Oct4-positive colonies (mouse)	~ 10.19%	**Pros**: Fast and high efficiency **Cons**: Use of retrovirus	Zhu et al. 2014
MEFs	Sall4, Jdp2, Glis1 and Esrrb	iPSCs	Oct4-positive colonies	~ 8%	**Pros**: High efficiency **Cons**: Use of retrovirus	Wang et al. 2023a, b
MEFs	VPA, CHIR99021, 616,452, Tranylcypromine, Forskolin, DZNep	iPSCs	Oct4-positive colonies	~ 0.04%	**Pros**: Safe **Cons**: Time-consuming and low efficiency	Hou et al. 2013
HEFs, hADSCs, hASFs	VPA, CHIR99021, 616,452 (Repsox), DZNep, PD0325901, tranylcypromine (Parnate), TTNPB, SAG, ABT-869, Y-27632, EPZ004777 or EPZ5676, ruxolitinib, JNK-IN-8, 5-azacytidine, BIRB796, dorsormorphin, SGC-CBP30, SB590885, vitamin C, UNC0379, IWP-2, UNC0224	iPSCs	Human ES-like colonies	~ 0.016%	**Pros**: Safe **Cons**: Time-consuming and low efficiency	Guan et al. 2022
HEFs, hADSCs, hASFs	VPA, CHIR99021, 616,452, DZNep, PD0325901, tranylcypromine, TTNPB, SAG, Y-27632, EPZ5676, ruxolitinib, JNK-IN-8, 5-Azacytidine, BIRB796, dorsormorphin, SGCCBP30, SB590885, vitamin C, VTP50469, AKT kinase inhibitor, SETD2-IN-1, 5-iodotubercidin, CX-4945	iPSCs	Human ES-like colonies	~ 1%−31%	**Pros**: Safe and high efficiency **Cons**: Time-consuming	Liuyang et al. 2023
MEFs	CHIR99021, 616,452, Forskolin, AM580, SR11237, CD3254, EPZ004777, VPA, CCT129202, R406, DMH1, BML-277, 5-aza-dC, AZD9291 mesylate, TSA, SGC0946, SGC-CBP30, citarinostat, TMP269, WS6, Vc, PD0325901	iPSCs	Oct4-positive colonies	~ 0.8%	**Pros**: Safe and fast-kinetics **Cons**: Low efficiency	Chen et al. 2023
MEFs	Ascl1, Brn2, Myt1l	Neurons	Tuj1-positive cells	~ 19.5%	**Pros**: Relative high efficiency **Cons**: Use of lentivirus	Vierbuchen et al. 2010
HEFs, HDFs	Ascl1, Brn2, Myt1l, NeuroD1	Neurons	Tuj1-positive cells	/	**Cons**: Low efficiency; Use of lentivirus	Pang et al. 2011
MEFs, human skin fibroblasts	Mash1, Nurr1, Lmx1a	Dopaminergic neurons	TH-positive cells	Mouse: ~ 21% Human: ~ 8%	**Cons**: Low efficiency; Use of lentivirus	Caiazzo et al. 2011
HFFs, HDFs	miR-9/9*−124, NeuroD2, Ascl1, Myt1l	Neurons	MAP2-positive cells	~ 5%	**Cons**: Low efficiency; Use of lentivirus	Yoo et al. 2011
HDFs	miR-9/9*−124, Ctip2, Dlx1, Dlx2, and Myt1l	Striatal medium spiny neurons	DARPP-32-positive cells	63%−72%	**Pros**: High efficiency and specificity **Cons**: Use of lentivirus	Victor et al. 2014
MEFs	Forskolin, ISX9, CHIR99021, SB431542	Neurons	Tuj1-positive cells	~ 90%	**Pros**: High efficiency; Safe **Cons**: Time-consuming	Li et al. 2015
Human fetal astrocytes	DAPT, CHIR99021, SB431542, and LDN193189	Glutamatergic neurons	NeuN-positive cells	~ 71%	**Pros**: High efficiency; Safe **Cons**: Time-consuming	Yin et al. 2019
Cardiac or dermal fibroblasts	Gata4, Mef2c, Tbx5	Cardiomyocytes	αMHC-positive cells	~ 20%	**Cons**: Use of lentivirus	Ieda et al. 2010
Murine cardiac fibroblasts	Gata4, Mef2c, Tbx5, Hand2	Cardiomyocytes	αMHC-positive cells	~ 19.7%	**Cons**: Use of retrovirus	Song et al. 2012
Murine cardiac fibroblasts	Polycistronic constructs containing Gata4, Mef2c, Tbx5	Cardiomyocytes	αMHC-positive cells	~ 15%	**Cons**: Use of retrovirus	Wang et al. 2015a, b
Murine TTFs	Gata4, Mef2c, Tbx5, Hand2, Akt1, Znf281	Cardiomyocytes	αMHC- and cTNT-double positive cells	∼28%	**Pros**: High efficiency **Cons**: Use of retrovirus	Zhou et al. 2017a, b
Murine TTFs	Gata4, Mef2c, Tbx5, Hand2, Akt1, Phf7	Cardiomyocytes	αMHC- and cTNT-double positive cells	∼20%	**Pros**: High efficiency **Cons**: Use of retrovirus	Garry et al. 2021
H9-derived fibroblasts, Human cardiac fibroblasts	Gata4, Mef2c, Tbx5, Tbx20	Cardiomyocytes	αMHC-positive cells	∼30%	**Pros**: High efficiency **Cons**: Use of lentivirus	Tang et al. 2022
HFFs	CHIR99021, A83-01, BIX01294, AS8351, SC1, Y27632, OAC2, SU16F, JNJ10198409	Cardiomyocytes	cTNT-positive cells	~ 7%	**Pros**: Safe **Cons**: Low efficiency	Cao et al. 2016
Murine fibroblasts, Murine cardiac fibroblasts	SB431542, Baricitinib, Gata4, Mef2c, Tbx5,	Cardiomyocytes	cTNT-positive cells	~ 37.2%	**Pros**: High efficiency; Safe **Cons**: Time-consuming	Tao et al. 2023
TTFs	Gata4, Hnf1a, Foxa3, inactivation of p19^Arf^	Hepatocytes	Number of epithelial colonies	/	**Cons**: Low efficiency; Use of lentivirus	Huang et al. 2011
MEFs, MDFs	Hnf4a plus Foxa1, Foxa2 or Foxa3	Hepatocytes	Albumin- and E-cadherin-double positive cells	85%	**Pros**: High efficiency **Cons**: Use of retrovirus	Sekiya and Suzuki 2011
HFFs	Foxa3, Hnf1a, Hnf4a	Hepatocytes	Albumin- and α−1-antitrypsin-double positive cells	20%	**Pros**: High efficiency **Cons**: Use of lentivirus	Huang et al. 2014
Human fibroblasts	Hnf1a, Hnf4a, Hnf6, Cebpa, Prox1, Atf5	Hepatocytes	Albumin-positive cells	~ 90%	**Pros**: High efficiency **Cons**: Use of lentivirus	Du et al. 2014
Human fibroblasts	Hnf4a, Hnf6a, Gata4, Foxa2, Hhex, c-Myc, p53-siRNA	Hepatocytes	Albumin-positive cells	~ 75%	**Pros**: High efficiency **Cons**: Use of lentivirus; Use of oncogene	Xie et al. 2019
MEFs	CHIR99021, RepSox, VPA, Parnate, TTNPB, Dznep plus a single transcription factor (Foxa1, Foxa2, or Foxa3)	Hepatocytes	Albumin- or E-cadherin-positive cells	E-cadherin-positive cells: 55.8%−61.8%; Albumin-positive cells: 24.2%−42.9%	**Pros**: High efficiency **Cons**: Use of retrovirus	Guo et al. 2017
MEFs, NSFs	CHIR99021, 616,452, Forskolin, AM580, EPZ004777, Forskolin, Vc	Hepatocytes	Albumin-positive cells	~ 15%	**Pros**: High efficiency **Cons**: Time-consuming	Bai et al. 2023

#### Small chemical-driven pluripotent reprogramming

Since the groundbreaking discovery of iPSCs by Yamanaka in 2006, substantial efforts have been made to identify small molecules that could replace some of Yamanaka factors used to reprogram somatic cells and/or enhance the generation of iPSCs from somatic cells (Yagi et al. 2024). For example, Huang and colleagues greatly boosted the efficiency of reprogramming mouse or human fibroblasts into iPSCs with the supplementation of valproic acid (VPA), a histone deacetylase (HDAC) inhibitor (Huangfu et al. 2008b, 2008a). Their proof-of-concept work demonstrated that chemicals could increase reprogramming efficiency and might be used to replace one or more reprogramming factors used for reprogramming. In a notable study led by Pei, the authors reported the administration of ascorbic acid (Vitamin C) enabled the rapid and efficient generation of high-quality iPSCs from mouse and human somatic cells (Esteban et al. 2010). Later, the authors found that Vitamin C enhanced the efficiency of cell reprogramming by modulating the enzymatic activity of ten-eleven translocation (Tet)-dependent DNA demethylases or histone H3 lysine-36 (H3K36) demethylases (Wang et al. 2011a; Chen et al. 2013a). In another noteworthy study, Hochedlinger and colleagues developed a rapid and synchronous reprogramming system by the combined supplementation of transforming growth factor β (TGF-β) antagonist (ALK5 inhibitor II) together with Wnt signaling agonist (CHIR99021) and Vitamin C (Bar-Nur et al. 2014). In a landmark study, Hou and co-workers reported the success of full chemical-driven cell reprogramming of fibroblasts towards iPSCs, which were termed as CiPSCs (Hou et al. 2013). The final cocktail identified in this study consisted of VPA, CHIR99021, E-616542, Tranylcypromine, Forskolin and DZNep (Hou et al. 2013). The chromatin accessibility dynamics during chemical reprogramming process was investigated by Cao and colleagues using an efficient two-step serum- and replating-free protocol for chemical-driven cell reprogramming (containing Vitamin C, BMP4, RepSox, BrdU, VPA, FSK, AM580, EPZ5676, DZNep, and SGC0946) (Cao et al. 2018). The authors found that chemical-driven cell reprogramming significantly differed from Yamanaka factor-based cell reprogramming approach in terms of reorganizing the genome architecture (Cao et al. 2018). Recently, Chen and co-workers refined the chemical recipe and developed a fast chemical reprogramming (FCR) system through large-scale chemical screenings (Chen et al. 2023). Using this robust chemical-driven reprogramming (FCR) system, they found that the chemical-driven acquisition of pluripotency was achieved through a developmentally diapause-like state (Chen et al. 2023). In a remarkable study, Guan and colleagues reported the successful generation of human chemically induced pluripotent stem cells that exhibit key features of embryonic stem cells from human somatic cells via chemical-driven reprogramming (Guan et al. 2022). The authors found that chemical cocktails (comprising CHIR99021, 616,452, TTNPB, Y-27632, SAG, ABT-869, JNKIN8, tranylcypromine, and 5-azacytidine) induced an intermediate plastic state, which was critical for acquiring cell pluripotency (Guan et al. 2022). Altogether, different combinations of small chemicals used in these aforementioned studies reveal the flexibility of chemical-driven pluripotent reprogramming.

#### The roles of biophysical cues in pluripotent reprogramming

**Fig. 2:**
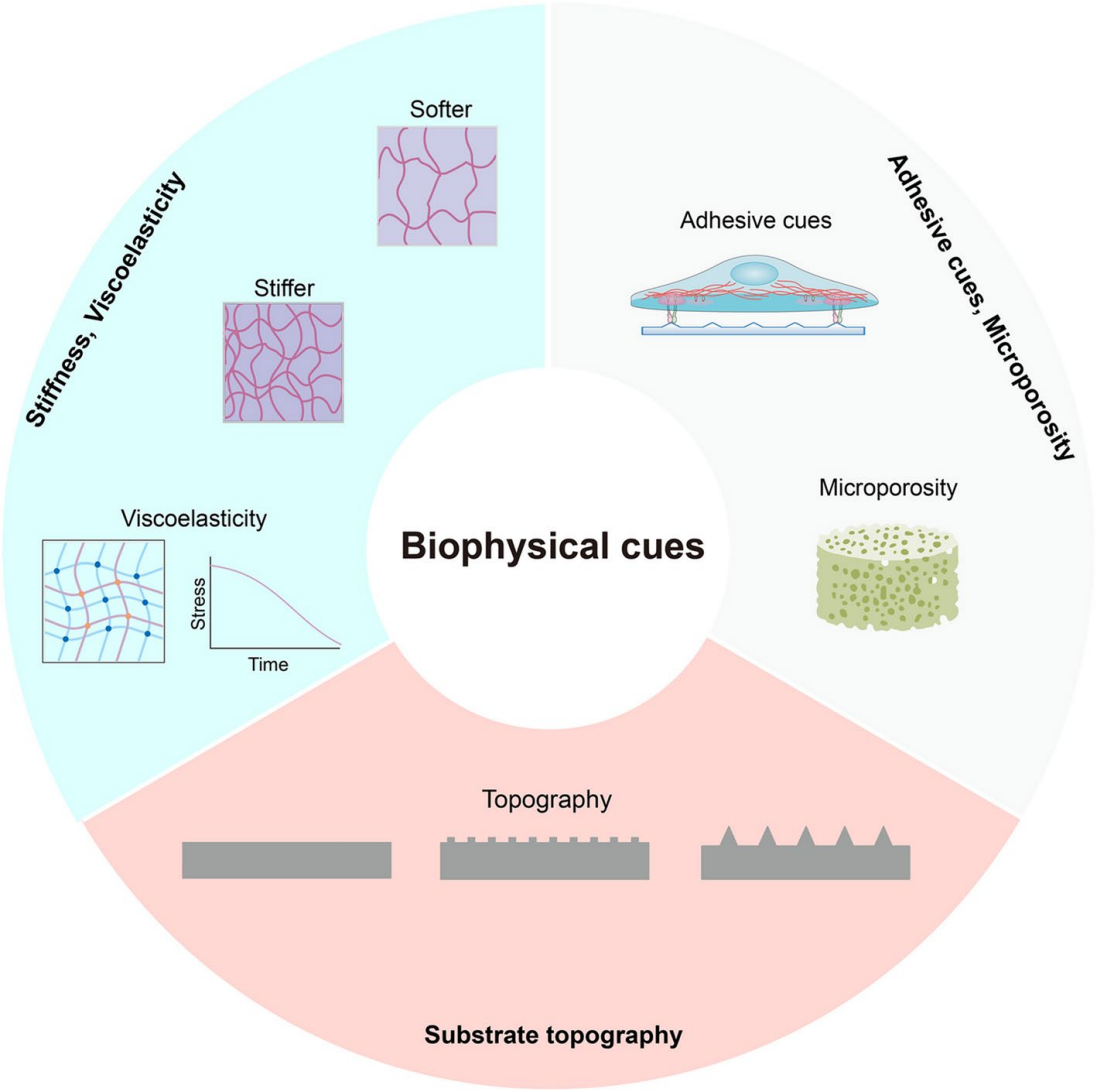
Different forms of biophysical cues from functional biomaterials. The important roles of biophysical cues, such as substrate stiffness, substrate topography, viscoelasticity, adhesive properties, or microporosity in cell reprogramming are increasingly recognized

Accumulating evidence has demonstrated that biophysical cues play important roles in cell reprogramming (Fig. 2). In a seminal study, Downing and colleagues described the enhanced generation of iPSCs via seeding mouse/human fibroblasts onto the micro-/nano-patterned microgrooves on the surface of cell-attached scaffolds (Downing et al. 2013). Mechanistically, the authors found that downregulated expression of HDAC2 and upregulated expression of WDR5 mediated by parallel microgrooves led to increased histone 3 (H3) acetylation and methylation (Downing et al. 2013). The biophysical cue-mediated modulation of epigenetic state allowed for the enhanced acquisition of pluripotency from somatic cells. Remarkably, Song and co-workers evaluated the effects of mechanical stimulation-mediated epigenetic priming on the outcomes of cell reprogramming (Song et al. 2022). They found that millisecond confinement of cells into microfluidic channels led to transient disassembly of the nuclear lamina, local detachment of lamina-associated domains in chromatin and a decrease of DNA methylation and histone H3 lysine 9 trimethylation, which boost the efficiency of cell reprogramming (Song et al. 2022). In a recent study, a super-hydrophobic microwell array chip (SMAR-chip) was developed to enable efficient cell reprogramming (Qu et al. 2023). The authors found that the elicitation of morphology change from 2D monolayers to 3D clusters by SMAR-chip overcame the barriers for reprogramming and promoted the transition from the initiation stage to the maturation stage of cell reprogramming. More recently, Zhu and colleagues demonstrated the robust reprogramming of different types of somatic cells into iPSCs by coordination of cell-reprogramming-inspired dynamically responsive hydrogel and phase separation of yes-associated protein (YAP) (Zhu et al. 2024). Notably, the authors observed that cell-reprogramming-inspired dynamically responsive hydrogel could faithfully sense metabolic remodeling and extracellular acidification during cell reprogramming, and respond by autonomously changing its mechanical properties (from softer to stiffer). The stiffening of the cell-reprogramming-responsive hydrogel elicited the nuclear translocation of YAP and the formation of YAP condensates with the appropriate timing during cell reprogramming, ensuring a faster and more efficient generation of iPSCs (Zhu et al. 2024). To sum, these aforementioned studies suggest that coordination of biochemical signals and biophysical cues is of great importance for achieving optimal outcomes of cell reprogramming.

### Methods for direct reprogramming

#### Transcription factor-mediated direct reprogramming

Reprogramming of somatic cells towards the pluripotent state (pluripotent reprogramming) has been regarded as the most representative example of modulating cell fate by the ectopic expression of transcription factors. The historical timeline of pluripotent reprogramming is reminiscent of direct reprogramming. Davis and colleagues demonstrated in their seminal study that the ectopic expression of myoblast-specific transcription factor (MyoD1) converted fibroblasts into myoblasts (Davis et al. 1987). Since then, different strategies for direct reprogramming have been developed to induce several cell types, such as neurons (Vierbuchen et al. 2010; Pang et al. 2011; Li et al. 2015; Jorstad et al. 2017), cardiomyocytes (Ieda et al. 2010; Qian et al. 2012; Cao et al. 2016), hepatocytes (Huang et al. 2011, 2014; Sekiya and Suzuki 2011), Sertoli cells (Liang et al. 2019), osteoblasts (Yamamoto et al. 2015), dendritic cells (Rosa et al. 2018), or natural killer cells (Kim et al. 2021). We will focus on neuronal reprogramming, cardiac reprogramming, and hepatic reprogramming as typical examples of direct reprogramming throughout this review.

##### Neuronal reprogramming

In two landmark studies led by Dr. Marius Wernig, the authors demonstrated the direct conversion of mouse/human fibroblasts to functional neurons through ectopic expression of a transcription factor combination comprising Ascl1, Brn2, and Myt1l (Vierbuchen et al. 2010; Pang et al. 2011) (Table 1). These induced neurons exhibited neuronal morphology, expressed neuron-specific genes, and displayed functional neuronal properties such as the generation of trains of action potentials and synapse formation (Vierbuchen et al. 2010; Pang et al. 2011). In 2014, Marius Wernig’s lab demonstrated that Ascl1 alone could directly convert mouse and human fibroblasts into induced neuronal cells (Chanda et al. 2014), suggesting ASCL1 is the key driver of neuronal reprogramming in different cell contexts. In a notable study, Caiazzo and colleagues reported the rapid and efficient conversion of prenatal and adult fibroblasts into functional dopaminergic neurons via the forced expression of a set of three transcription factors—*Mash1*, *Nurr1* and *Lmx1a* (Caiazzo et al. 2011). The authors found that the derived neurons were similar to brain dopaminergic neurons in morphology and gene expression signatures, and exhibited dopamine release and pacemaker activity (Caiazzo et al. 2011). Pure and functional cholinergic neurons have been efficiently converted from human fetal fibroblasts with NGN2, SOX11 and two chemical molecules: forskolin and dorsomorphin (Liu et al. 2013). The human induced cholinergic neurons showed motor neuron-like features, including morphology, gene expression profiles and the formation of functional neuromuscular junctions (Liu et al. 2013). The generation of GABAergic neurons from non-neural astrocytes has also been achieved via co-expression of NeuroD1 and Dlx2 (Wu et al. 2020). The derivation of GABAergic neurons from mature NG2 glial cells required the co-expression of Ascl1, Lmx1a, and Nurr1 (Wang et al. 2021a). Remarkably, Yoo and colleagues described the conversion of human fibroblasts into mature neurons using a cocktail incorporating miR-9/9*−124, ASCL1, MYT1L and NEUROD2, demonstrating the neurogenic activity of the genetic circuitry involving brain-enriched miR-9/9*−124 (Yoo et al. 2011). One of downstream targets of miR-124 is polypyrimidine-tract-binding protein (PTBP1), which is essential to normal brain development (Xue et al. 2013). Repression of PTBP1 has been documented, in an elegant study, to be sufficient to directly convert fibroblasts towards functional neurons (Xue et al. 2013). Later studies corroborated this finding by demonstrating that the repression of PTBP1 enabled the successful neuronal reprogramming from oligodendrocytes (Weinberg et al. 2017), astrocytes (Qian et al. 2020), or gial cells (Zhou et al. 2020). The critical role of neuron-specific alternative splicing in direct conversion of human skin fibroblasts into neurons was revealed in a recent study, which demonstrated that knockdown of PTBP2 significantly enhanced ASCL1-mediated neuronal reprogramming of human fibroblasts (Zhu et al. 2023a)*.* More recently, a miRNA-mediated, 3D culture-based neuronal reprogramming system was developed to convert patient-derived fibroblasts into cortical neurons (Sun et al. 2024). These patient-derived cortical neurons could faithfully recapitulate Aβ deposition, tauopathy, and neurodegeneration phenotypes of late-onset Alzheimer’s disease (LOAD) (Sun et al. 2024), enabling therapeutic drug screening in clinically more relevant disease model.

##### Cardiac reprogramming

In a seminal study, Ieda and co-workers demonstrated the rapid and efficient conversion of cardiomyocyte-like cells from postnatal cardiac and dermal fibroblasts via the forced expression of three heart lineage-specific transcription factors, Gata4, Mef2c, and Tbx5 (Ieda et al. 2010) (Table 1). The induced cardiomyocyte-like cells were similar to neonatal cardiomyocytes in terms of global gene expression profile, and could contract spontaneously (Ieda et al. 2010). Two years later, the same group reported in vivo cardiac reprogramming for the first time and explored the regenerative potential in myocardial infarction (Qian et al. 2012). Specifically, the authors achieved the conversion of non-myocytes to induced cardiomyocytes in vivo by local delivery of Gata4, Mef2c, and Tbx5 into non-myocytes of myocardial infarction mice (Qian et al. 2012). The induced cardiomyocytes were found to improve cardiac function of myocardial infarction mice. In another notable study, Song and colleagues reported the improved function of injured heart through the direct conversion of non-myocytes to cardiomyocytes in vivo, which was achieved by retroviral delivery of Gata4, Hand2, Mef2c, and Tbx5 into non-myocytes (Song et al. 2012). However, the relative low conversion rate of direct cardiac reprogramming prompted efforts to optimize the reprogramming factor cocktail and delivery method. To this end, a complete set of polycistronic constructs were generated to include all six possible splicing orders of cardiac reprogramming factors (comprising Gata4 (G), Mef2c (M), and Tbx5 (T)) in the same construct with identical 2A peptides (Wang et al. 2015a). The authors demonstrated that difference in protein stoichiometry of G, M, and T was sufficient to exert a significant influence on the efficiency and quality of cardiac reprogramming (Wang et al. 2015a). The optimal stoichiometry of G, M, and T, defined by higher expression level of Mef2c and lower expression levels of Gata4 and Tbx5, was correlated with more efficient and complete conversion of fibroblasts into cardiac myocyte–like cells (Wang et al. 2015a).

Modulation of relevant signaling pathways has also been well documented to improve the efficiency of cardiac reprogramming. For example, inhibition of TGF-β signaling has been demonstrated to enhance the conversion efficiency of cardiac reprogramming (Zhao et al. 2015). The combinatorial inhibition of TGF-β and WNT signaling with SB431542 and XAV939 was found, by Mohamed and colleagues, to improve the efficiency of GMT (Gata4, Mef2c, and Tbx5)-mediated cardiac reprogramming by eightfold (Mohamed et al. 2017). The influence of proinflammatory signaling on cardiac reprogramming has been interrogated by Ieda and co-workers. The authors identified diclofenac sodium, a non-steroidal anti-inflammatory drug, dramatically augmented the efficiency of cardiac reprogramming mediated by *Gata4*, *Mef2c*, and *Tbx5* (GMT) or GMT plus *Hand2* (GHMT) (Muraoka et al. 2019). The potential influence of Notch signaling on cardiac reprogramming has been investigated by Olson and colleagues. The authors found that pharmacological inhibition of NOTCH signaling by DAPT enhanced cardiac reprogramming mediated by GATA4, HAND2, MEF2C, and TBX5. Mechanistically, DAPT was found to increase activity of the transcription factor MEF2C and bind of MEF2C to the promoters of cardiac signature genes (Abad et al. 2017). Notably, overexpression of autophagy-related 5 (Atg5) was reported, in a recent study, to improve the efficiency of cardiac reprogramming (Wang et al. 2020a), demonstrating the requirement of autophagy in cardiac reprogramming. Remarkably, the polycomb complex gene *Bmi1* was reported to be an epigenetic barrier to cardiac reprogramming in a shRNA-based loss-of-function screening (Zhou et al. 2016). Knocking down of *Bmi1* led to the improved efficiency of cardiac reprogramming (Zhou et al. 2016). This proof-of-concept study demonstrated the feasibility of RNAi-mediated functional screening to identify epigenetic barriers to cardiac reprogramming (Zhou et al. 2016). The addition of other transcription factors, such as HAND2, AKT1, ZNF281, PHF7, and TBX20 to the core reprogramming cocktail (Gata4, Mef2c, and Tbx5) has been demonstrated to significantly improve the efficiency and quality of cardiac reprogramming (Song et al. 2012; Zhou et al. 2015, 2017a; Garry et al. 2021; Tang et al. 2022). Surprisingly, miRNA-mediated genetic circuits have also been implicated in cardiac reprogramming. For example, Jayawardena and colleagues presented evidence that direct conversion of fibroblasts into cardiomyocyte-like cells could be achieved by a combination of four miRNAs (miR-1, miR-133, miR-208, and miR-499) both in vitro and in vivo (Jayawardena et al. 2012). The miRNA-induced cardiomyocyte-like cells were endowed with functional properties characteristic of cardiomyocytes such as L-type channel expression, spontaneous calcium oscillations in response to depolarization, and contractility (Jayawardena et al. 2012). Interestingly, Wang and colleagues demonstrated in a recent study that the efficient cardiac reprogramming was driven by the combination of MEF2C and ASCL1, a well-known neuron-specific transcription factor (Wang et al. 2022a). Mechanistically, the authors found that MEF2C mediated the shift in ASCL1 binding away from neuronal genes toward cardiac genes, guiding their co-operative epigenetic and transcription activities (Wang et al. 2022a). Zhou and co-workers described the efficient conversion of human cardiac fibroblasts into cardiomyocytes using an optimized, clinically applicable adeno-associated virus (AAV) capsid encoding MYOCD, ASCL1 along with miR-133 and the miR-208 (Zhou et al. 2023). In a recent study, Xie and colleagues uncovered Ybx1 as a critical barrier to the induction of cardiomyocyte-like cells from non-myocytes, demonstrating the involvement of RNA-binding protein in the cardiac reprogramming process (Xie et al. 2023b). More recently, functional screening of small chemicals have identified SB431542 + Baricitinib (Tao et al. 2023), or vitamin C (Fang et al. 2024), to significantly increase the efficiency of cardiac reprogramming in GMT-overexpressing fibroblasts through the inhibition of inhibiting Alk5 and Tyk2, or reactive oxygen species, respectively.

##### Hepatic reprogramming

Several studies have reported direct conversion of somatic cells into hepatocytes via the ectopic expression of liver lineage-specific transcription factors in somatic cells (Table 1). Two independent groups initially reported the direct conversion of mouse fibroblasts to functional hepatocyte-like cells (iHeps) (Huang et al. 2011; Sekiya and Suzuki 2011). Huang and colleagues used the combination of Gata4, Hnf1α and Foxa3, and inactivation of p19^Arf^ (Huang et al. 2011), while Sayaka Sekiya and Atsushi Suzuki identified another set of transcription factors comprising Hnf4a plus Foxa1, Foxa2 or Foxa3 (Sekiya and Suzuki 2011). These induced hepatocyte-like cells (iHeps) exhibited typical epithelial morphology, expressed hepatic-signature genes, had hepatocyte-specific features, and repopulated damaged hepatic tissue after intrasplenically injected into fumarylacetoacetate-hydrolase-deficient (*Fah*^−/−^) mice and rescued almost half of recipient mice from death (Huang et al. 2011; Sekiya and Suzuki 2011). A few years later, Dr. Hui’s group reported the generation of functionally mature human-induced hepatocytes (hiHeps) directly from human fetal fibroblasts, adult fibroblasts, and adult adipose tissue-derived mesenchymal stem cells by lentiviral-mediated delivery of *FOXA3*, HNF1A, and *HNF4A* (Huang et al. 2014). The authors observed that hiHeps were highly similar to human primary hepatocytes in terms of gene expression pattern, hepatic phenotypes and functions (Huang et al. 2014). Importantly, hiHeps were able to restore liver function and prolong survival after being transplanted into mice with concanavalin-A-induced acute liver failure or fatal metabolic liver disease due to fumarylacetoacetate dehydrolase (Fah) deficiency (Huang et al. 2014). Notably, Du and colleagues reported the reproducible generation of human hiHeps with drug-metabolizing functions from human embryonic fibroblasts using a combination of hepatic-lineage transcription factors *HNF1A*, *HNF4A*, and *HNF6* together with the maturation factors *ATF5*, *PROX1*, and *CEBPA* (Du et al. 2014). In functional assay, hiHeps were found to robustly repopulate up to 30% of the liver of Tet-uPA/Rag2^−/−^/γc^−/−^ mice and were functional in vivo (Du et al. 2014). In vivo hepatic reprogramming was demonstrated by Sharma and colleagues in a noteworthy study, in which simultaneous expression of transcription factors FOXA3, GATA4, HNF1A, and HNF4A converted mouse myofibroblasts into cells with hepatocyte phenotype and functional properties (Song et al. 2016). Importantly, in vivo hepatic reprogramming induced by FOXA3, GATA4, HNF1A, and HNF4A ameliorated liver fibrosis in carbon tetrachloride-injected mice for 8 weeks (Song et al. 2016). Notably, Xie and colleagues described a two-step hepatic reprogramming strategy to generate hiHeps abundantly and functionally (Xie et al. 2019). The generation of functional hiHeps lies in the intermediate generation of expandable hepatic progenitors during the two-step hepatic reprogramming via combined genetic and chemical approaches (Xie et al. 2019). The induced hepatic progenitors reported in this study could serve as a stable source to generate large quantities of hepatocytes and meet the quantitative demand of biomedical applications of human hepatocytes. More recently, Clustered regularly interspaced short palindromic repeats activator (CRISPRa)-mediated expression of endogenous Gata4 and Foxa3 was found to be sufficient to successfully induce the direct conversion of MEFs into functional iHeps with capacity of drug metabolism and glycogen storage (Li et al. 2024a), representing a promising strategy to treating liver fibrosis caused by hepatic diseases.

#### Small chemical-driven direct reprogramming

In comparison with transcription factor-mediated direct reprogramming, small chemical-driven direct reprogramming offers an alternative way that can manipulate cell fates in a simple, safe, precise and highly controllable manner. Small chemicals are non-integrative to the genome, easily standardized and manufactured, and cost-effective, rendering small chemical-driven direct reprogramming as a promising avenue for the generation of cell products for therapeutic applications (Wang et al. 2023b). Desired cell types including neurons (Li et al. 2015; Yin et al. 2019; Ma et al. 2021), cardiomyocytes (Fu et al. 2015; Cao et al. 2016; Huang et al. 2018; Tao et al. 2023) and hepatocytes (Guo et al. 2017; Bai et al. 2023) have been derived using small chemical-driven direct reprogramming.

##### Neuronal reprogramming

Increasing evidence suggests that small chemicals can modulate neuronal reprogramming (Table 1). In a notable study, the authors described a neuronal reprogramming method to efficiently convert fibroblasts into functional neurons with a cocktail of small molecules (Forskolin, ISX9, CHIR99021, and I-BET151) (Li et al. 2015). The chemically induced neurons possessed neuronal properties with respect to gene expression pattern and generation of action potentials and formed functional synapses (Li et al. 2015). Full chemical-driven neuronal reprogramming of human fibroblasts into glutamatergic neurons was reported by Hu and colleagues, who identified a chemical cocktail of seven small molecules (Valproic acid, CHIR99021, Repsox, Forskolin, SP600125, GO6983, and Y-27632) (Hu et al. 2015). The authors found that human chemical-induced neuronal cells resembled hiPSC-derived neurons and human induced neurons in terms of morphology, neural-specific gene expression profiles, and electrophysiological properties (Hu et al. 2015). Brain astrocytes, one type of neural stromal cells have also been chemically reprogrammed to functional neurons upon that sequential exposure to a cocktail of nine chemicals (Zhang et al. 2015). The chemically converted human neurons were found to be functional, as evidenced by synaptic events and synchronous burst activities (Zhang et al. 2015). Follow-up studies converted human fetal astrocytes into neurons via another set of small chemical cocktail, including DAPT, CHIR99021, SB431542, and LDN193189 (Yin et al. 2019). This study showed that small chemical-based, synergistic modulation of Notch, glycogen synthase kinase 3, transforming growth factor β, and bone morphogenetic protein pathways was sufficient for astrocyte-to-neuron conversion (Yin et al. 2019). Recently, a combination of small chemicals (Forskolin, ISX9, CHIR99021, I-BET151, and Y-27632) has been identified to directly convert endogenous astrocytes into functional neurons in the adult mouse brain (Ma et al. 2021). These chemically induced neurons exhibited neuronal morphology, expressed neuronal-specific markers, and acquired electrophysiological functions (Ma et al. 2021). However, the dosage of small chemicals used for in vivo neuronal reprogramming is much higher than that of in vitro due to the influence of blood brain barrier (Ma et al. 2021; Wan and Ding 2023), which limit the clinical applications of small chemicals in vivo. Therefore, future research and explorations are needed to determine the optimal combinations/dosage of chemicals in different scenarios.

##### Cardiac reprogramming

Increasing evidence has reported the role of small chemical cocktails in direct conversion of fibroblasts into cardiomyocyte-like cells (Table 1). The initial report of chemical-driven cardiac reprogramming was led by Xie and co-workers, demonstrating that the generation of automatically beating cardiomyocyte-like cells from mouse fibroblasts in vitro and in vivo using only chemical cocktail (CRFVPTM: CHIR99021, RepSox; Forskolin, VPA, Parnate, TTNPB, Rolipram) (Fu et al. 2015). In an elegant study, Cao and colleagues developed another chemical cocktail (including CHIR99021, A83-01, BIX01294, AS8351, SC1, Y27632, OAC2, SU16F and JNJ10198409) to enable the completion of cardiac reprogramming from fibroblasts (Cao et al. 2016). The chemically induced cardiomyocyte-like cells resembled human cardiomyocytes in their transcriptome, epigenetic, and electrophysiological properties, were functionally contracted (Cao et al. 2016). Recently, Xie and colleagues investigated the effect of the chemical cocktail CRFVPTM in inducing in situ cardiac reprogramming of fibroblasts at the whole-organ level and providing a new source of naturally incorporated cardiomyocytes to help heart regeneration (Huang et al. 2018). More recently, Tao and co-workers identified ALK5 and JAKs/TYK2 inhibitors (2C) to greatly enhance the efficiency of cardiac reprogramming mediated by Gata4, Mef2c, and Tbx5 (Tao et al. 2023). The authors found that cardiomyocyte-like cells induced by 2C treatment possessed enhanced functional properties, which resembled those of adult cardiomyocytes (Tao et al. 2023).

##### Hepatic reprogramming

Small chemicals have been applied to improve the efficiency of hepatic reprogramming (Table 1). For example, Guo and colleagues reported transdifferentiation of mouse fibroblasts into hepatocyte-like cells (iHeps) using a single transcription factor (Foxa1, Foxa2, or Foxa3) plus a chemical cocktail (comprising CHIR99021, RepSox, VPA, Parnate, TTNPB, and Dznep) (Guo et al. 2017). iHeps could exert hepatic function in vivo, reconstitute the damaged hepatic tissues, and prolong the life of the fumarylacetoacetate hydrolase-deficient (*Fah*^−/−^) mice after 2-(2-nitro-4 trifluoro-methylbenzyol)−1,3-cyclohexanedione withdrawal (Guo et al. 2017). In another notable study, Lim and colleagues demonstrated the combination of *Hnf1α* and two chemicals (A-83–01, CHIR99021) is sufficient to induce hepatic reprogramming to generate functional iHeps (Lim et al. 2016). Full chemical-driven hepatic reprogramming to generate hepatocyte-like cells (CiHeps) has been reported by Bai and co-workers in a recent study (Bai et al. 2023). The hepatic reprogramming of mouse fibroblasts into CiHeps was achieved via early-stage exposure to one set of chemical cocktails (containing CHIR99021, 616,452, Forskolin, AM580, and EPZ004777) followed by late-stage exposure to another set of chemical cocktails (containing CHIR99021, 616,452, Forskolin, and Vc) (Bai et al. 2023). CiHeps were similar to primary hepatocytes in terms of gene expression profiles, drug metabolic activity in vitro and liver repopulation in *Fah*^−/−^ mice in vivo (Bai et al. 2023). Small chemical optimization strategy described in this study may guide to full chemical-driven hepatic reprogramming of human fibroblasts into functional hepatocyte-like cells in the near future.

#### The role of biophysical cues in direct reprogramming

##### Neuronal reprogramming

Accumulative evidence has established the concept that biophysical cues (stiffness, substrate topography, and extracellular matrix) are critical modulators of direct reprogramming (Fig. 2). The effects of various topographies on the outcome of neuronal reprogramming were investigated by Kulangara and colleagues (Kulangara et al. 2014). The authors found that the final purity and the conversion efficiency of induced neurons were increased on micrograting scaffold, demonstrating the cell-topography interactions could influence neuronal reprogramming with respect to gene expression pattern, neurite branching, and reprogramming efficiency (Kulangara et al. 2014). Yoo and co-workers compared the outcomes of dopaminergic neuronal reprogramming of fibroblasts cultured on flat, microgrooved, or nanogrooved substrates (Yoo et al. 2015). The expression level of dopaminergic neuron markers, the maturity of dopaminergic neuronal phenotypes, and the conversion efficiency were enhanced mostly on nanogrooved substrate (Yoo et al. 2015), indicating that nano-topographical cues could serve as an efficient stimulant for direct reprogramming.

In a notable study, Jin et al. reported a reproducible and scalable neuronal reprogramming system, which was facilitated by 3D-brain extracellular matrix (BEM) decellularized from human brain tissue (Jin et al. 2018). 3D BEM hydrogels recapitulated brain tissue-specific microenvironments, substantially promoting neuronal conversion and potentiating the functional recovery of the animals (Jin et al. 2018). Of interest, a robust method to directly reprogram fibroblasts into neurons is reported via disruption of cytoskeletal architectures followed by exposure to neurogenesis-promoting factors (He et al. 2022). Mechanistically, the authors observed that cytoskeletal disruption induced cell softening, elicited YAP/TAZ nuclear export and induced a neuron-like state, which could be further converted into mature neurons upon the treatment with a neurogenic compound, ISX-9 (He et al. 2022). The influence of the stiffness of substrates on neuronal reprogramming has been recently interrogated by Xu and colleagues (Xu et al. 2022). Soft substrates were found to markedly improve the conversion efficiency and kinetics of neuronal reprogramming, bypassed the need to co-culture with glial cells during neuronal conversion, yielded more glutamatergic neurons with electrophysiological activity (Xu et al. 2022). More recently, Song and co-workers demonstrated the biphasic dependence of neuronal reprogramming on matrix stiffness (Song et al. 2024). The authors found that optimal neuronal reprogramming was achieved on surfaces of intermediate stiffness (~ 20 kPa), which induced a higher amount of cofilin and G-actin, a higher percentage of nucleus-localized histone acetyltransferase (HAT) enzymes, a more open chromatin structure, thereby increasing histone H3 acetylation that is conducive to neuronal reprogramming (Song et al. 2024).

##### Cardiac reprogramming

The effects of biophysical cues on cardiac reprogramming have also been extensively investigated. For example, Sia and colleagues evaluated the influence of microgrooved substrate on cardiac reprogramming (Sia et al. 2016). Mkl1 was identified as the mediator of the promoting effects of micro-topographical cues on cardiac reprogramming of fibroblasts infected with GATA4, MEF2 and TBX5 (Sia et al. 2016). The influence of substrate rigidity and mechanotransduction on cardiac reprogramming have been recently explored by seeding GHMT-transduced fibroblasts within Matrigel-based hydrogel (Kurotsu et al. 2020). The authors found that soft hydrogel comparable with native myocardium promoted the efficient generation of cardiomyocyte-like cells with higher quality (Kurotsu et al. 2020). Mechanistic study revealed that softer hydrogel enhanced cardiac reprogramming through combined inhibition of fibroblast program and integrin-Rho/ROCK-actomyosin-YAP/TAZ signaling cascade (Kurotsu et al. 2020). More recently, a 3D microenvironment reconstituted with decellularized heart extracellular matrix (3D-HEM) was fabricated by Jin and colleagues (Jin et al. 2022). The authors found that 3D-HEM could enhance chemical-driven cardiac reprogramming of fibroblasts into cardiomyocytes with a higher rate of expression of cardiac-specific markers, sarcomeric organization, contractility, and synchronization than the use of 2D substrates (Jin et al. 2022). In functional assay, transplantation of cardiomyocyte-like cells reprogrammed within 3D-HEM improved hemodynamic cardiac contractibility, reduced fibrosis and improved cardiac function in a rat model of myocardial infarction (Jin et al. 2022). We anticipate that the combination of cell reprogramming technology with state-of-the-art multifunctional biomaterials in the near future will enable more precise control of cell reprogramming to yield cells of interests with higher efficiency and quality.

## The molecular mechanisms governing cell reprogramming

### The molecular mechanisms governing pluripotent reprogramming

**Fig. 3:**
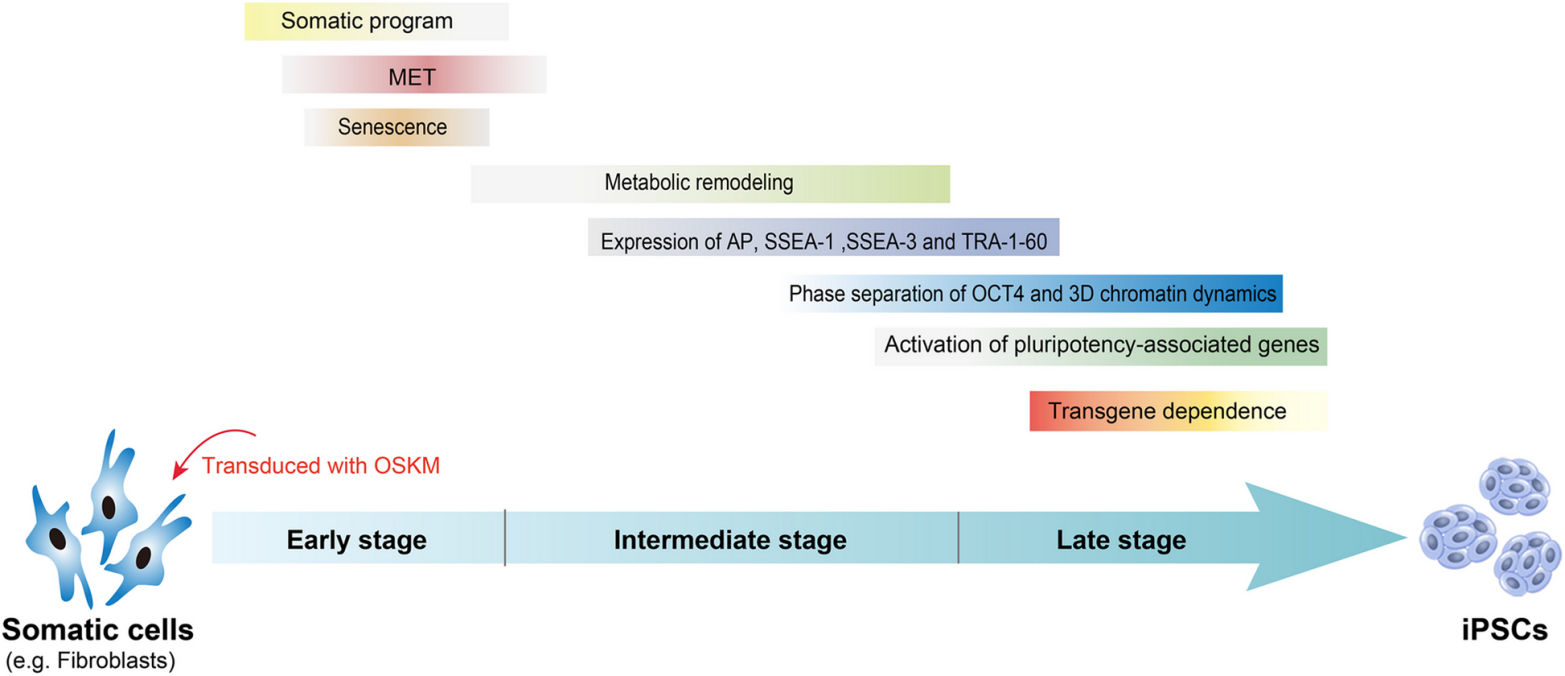
Key molecular events that occur in the process of pluripotent reprogramming. A summary of cellular and molecular events that occur during the generation of induced pluripotent stem cells (iPSCs) from fibroblasts. MET: mesenchymal-to-epithelial-transition; OKSM: OCT4, KLF4, SOX2, c-MYC

Since the initial report of the generation of iPSCs by Takahashi and Yamanaka in 2006, researchers have made longstanding efforts to investigate the mechanism of pluripotent reprogramming. The “elite model” supposes that only a small percentage of donor cells are endowed with the ability to become iPSCs, while the “stochastic model” proposes that most, if not all, donor cells have the potential to be competent for reprogramming (Yamanaka 2009). The “stochastic model” is supported by evidence that almost all donor cells are competent for generating iPSCs upon continued growth and the sustained expression of reprogramming factors (Hanna et al. 2009). The process of pluripotent reprogramming of somatic cells into induced pluripotent stem cells (iPSCs) is broadly categorized into a stochastic early-phase, an intermediate phase, and a deterministic late-phase (Cerneckis et al. 2024; Buganim et al. 2013). The molecular events for successful pluripotent reprogramming mainly include (Fig. 3): (1) the silence of somatic-signature genes (Cerneckis et al. 2024), (2) a proliferative outbreaks and the bypass of cellular senescence (Li et al. 2009; Banito et al. 2009), (3) the mesenchymal-to-epithelial transition (MET) (Li et al. 2010; Samavarchi-Tehrani et al. 2010), (4) a metabolic remodeling from oxidative phosphorylation to glycolysis-based metabolism (Zhang et al. 2016; Zhu et al. 2024), (5) the activation of early pluripotency-associated genes (such as alkaline phosphatase and SSEA-1 in mouse; alkaline phosphatase, SSEA3 and TRA-1–60 in human) (Xu et al. 2016); (6) the activation of core pluripotency network (Xu et al. 2016); (7) the silencing of exogenous transgenes (Apostolou and Hochedlinger 2013). Previous studies have established the notion that pluripotent reprogramming is a complex, coordinated, and dynamic process featured by transcriptional, epigenetic, metabolic or proteomic remodeling (Krause et al. 2016; Chen 2020; Yagi et al. 2024; Cerneckis et al. 2024). The investigations using single-cell technologies have revealed latent reprogramming trajectories during the reprogramming process (Guo et al. 2019; Schiebinger et al. 2019). The recent findings demonstrated that the involvement of phase separation-mediated biomolecular condensation and 3D chromatin architecture dynamics in pluripotent reprogramming (Fig. 3), adding novel insights on the mechanisms behind pluripotent reprogramming (Wang et al. 2021d; Ling et al. 2022; Zhu et al. 2024).

#### Transcription factors

The pluripotent reprogramming process is featured by the reactivation of pluripotency-associated genes, given that pluripotency-associated genes are localized in condensed chromatin regions in somatic cells. OCT4, SOX2 and KLF4 (OSK for short) have been defined as a subset class of transcription factors, referred to as “pioneer transcription factors” (Soufi et al. 2012). The pioneer activity of ectopically expressed OSK endowed them to bind globally and promiscuously with distal regulatory elements in the early stage of pluripotent reprogramming, evict somatic cell-signature transcription factors away from somatic enhancers, consequently silence of the enhancers of somatic cell-specific genes (Soufi et al. 2012, 2015). As the cell reprogramming process moves on, OSK engage in a sequential manner with the enhancers and promoters of pluripotency-associated genes, followed by opening of the local chromatin structure, thus enabling regulatory events required for gene regulation (Chronis et al. 2017). Sox2, in addition to the pioneer activity, has even been identified to induce DNA demethylation and overcome repressive epigenetic marks (Vanzan et al. 2021). Detailed analysis of the binding kinetics between Oct4 and nucleosome demonstrates the requirement of stable interactions of Oct4 with nucleosomes in maintaining chromatin accessibility during cell fate transitions and pluripotency maintenance (Roberts et al. 2021). A recent study has revealed the dynamic role for Klf4 in directing enhancer-promoter contacts and chromatin topological reorganization, which is crucial for successful pluripotent reprogramming (Di Giammartino et al. 2019). More recently, ZFP266 was identified to exert negative influence of chromatin opening mediated by OSK (Kaemena et al. 2023). ZFP266 is able to bind with Short Interspersed Nuclear Elements (SINEs) adjacent to the binding sites of OSK during pluripotent reprogramming, and impedes chromatin opening (Kaemena et al. 2023). c-Myc greatly enhances the efficiency of reprogramming by mediating the initial downregulation of somatic-specific genes and by igniting the biosynthetic module, which are crucial for deterministic reprogramming towards pluripotency (Sridharan et al. 2009; Zviran et al. 2019). Substantial efforts have been made to identify additional transcription factors, such as Dppa2/4 (Hernandez et al. 2018), c-Jun (Liu et al. 2015), AP-1 (Markov et al. 2021), NKX3-1 (Mai et al. 2018), as important modulators in pluripotent reprogramming. Intriguingly, ectopic expression of a group of lineage specifiers rather than pluripotency-associated transcription factors has been demonstrated to trigger iPSC induction from somatic cells (Montserrat et al. 2013; Shu et al. 2015), indicating that simultaneous inhibition of the major pathway governing mesendodermal and ectodermal differentiation makes somatic cells competent to become iPSCs. Together, these findings demonstrate that reprogramming transcription factors are drivers of pluripotent reprogramming through early shutdown of the somatic program and progressive induction of pluripotent program.

#### Chromatin remodeling and histone modifications

The first report of iPSCs by Shinya Yamanaka has spurred longstanding efforts to identify facilitators and roadblocks to pluripotent reprogramming, achieving a more efficient and faster reprogramming. It has been demonstrated that the generation of a multiplicity of specialized cellular types from pluripotent stem cells during embryonic development is accompanied with chromatin compaction, which is beneficial to safeguard cell identity (Zhu et al. 2013). Therefore, chromatin decompaction and remodeling is crucial for the acquisition of pluripotency from somatic cells. To support this notion, chromatin assembly factor-1 (CAF-1) complex was identified as a potent barrier of the pluripotent reprogramming process (Cheloufi et al. 2015). Mechanistically, inhibition of CAF-1 function rendered the distal regulatory element more accessible to Sox2, leading to increased binding of Sox2 to pluripotency-specific targets and activation of pluripotency-associated genes and facilitating the induction of iPSCs (Cheloufi et al. 2015). Interestingly, overexpression of ASF1A, another histone chaperone, demonstrated the promoting effects on the generation of iPSCs (Gonzalez-Muñoz et al. 2014). Nucleosome Remodeling and Deacetylase (NuRD) complex was found to play important roles during induction of pluripotency (dos Santos et al. 2014). Overexpression of Mbd3/NuRD facilitated pluripotent reprogramming through context-dependent regulation of gene expression (dos Santos et al. 2014). Chromatin-remodeling components of the BAF complex have been identified as enhancers of pluripotent reprogramming by facilitating enhanced Oct4 binding to target promoters (Singhal et al. 2010).

Histone methyltransferases/demethylases and histone acetyltransferases/deacetylases are well-known histone-modifying enzymes, which regulate histone marks and chromatin structure (Onder et al. 2012). Histone-modifying enzymes have been identified to play critical yet complex roles in pluripotent reprogramming (Onder et al. 2012). The histone methyltransferase EZH2 has been demonstrated to impose positive influence on pluripotent reprogramming, which was achieved via silencing of the expression of somatic cell-signature genes (Onder et al. 2012). In contrast to the positive role of EZH2 in pluripotent reprogramming, DOT1L was proved to act as an inhibitory modulator to the pluripotent reprogramming process, suggesting the involvement of histone H3K79 methylation in the reprogramming process (Onder et al. 2012). In addition to DOT1L, H3K4 methyltransferase MLL1 (Hörmanseder et al. 2017), H3K4 methylation effector Wdr5 (Ang et al. 2011), H3K9me3 demethylase KDM4A (Chen et al. 2013b), and H3K27 demethylase UTX (Mansour et al. 2012) have been identified as facilitators or barriers to the induction of iPSCs, highlighting the influence of H3K4me3, H3K9me3 or H3K27me3 histone modifications on the reprogramming process. Intriguingly, macroH2A and H3K27me3 are co-localized at UTX target genes (Gaspar-Maia et al. 2013). Loss of macroH2A2 yielded the higher percentage of SSEA1-positive cells and more iPSC colonies during pluripotent reprogramming, demonstrating histone variant (macroH2A2) act as a roadblock to reprogramming of somatic cells into iPSCs (Gaspar-Maia et al. 2013). Glis1 was recently identified as a positive regulator of global histone remodeling during the reprogramming process (Li et al. 2020). Mechanistically, Glis1 was observed to bind directly to and open chromatin at glycolysis gene loci, resulting in the upregulation of glycolysis-signature genes, followed by the elevated levels of cellular acetyl-CoA and lactate (Li et al. 2020). These events eventually led to the deposition of H3K27 acetylation and H3K18 lactylation at pluripotency gene loci, thereby facilitating pluripotent reprogramming (Li et al. 2020).

#### DNA methylation

Another form of epigenetic changes during pluripotent reprogramming is DNA methylation, which occur at later stage of the pluripotent reprogramming process (Polo et al. 2012). Interestingly, de novo DNA methylation mediated by Dnmt3a and Dnmt3b is dispensable for reprogramming of somatic cells into iPSCs (Pawlak and Jaenisch 2011). Downregulation of maintenance methyltransferase Dnmt1 in intermediate partially reprogrammed cells rendered their transition towards mature iPSCs (Mikkelsen et al. 2008), suggesting the supportive role of passive demethylation in iPSC formation. Another form of DNA demethylation is active demethylation, mediated by the Ten-eleven translocation (TET) family enzymes: TET1, TET2 and TET3. TET proteins are 5mC hydroxylases, which mediate the progressive conversion of 5-methylcytosine (5mC) to 5-hydroxymethylcytosine (5hmC), 5-formylcytosine (5fC), or 5-carboxylcytosine (5caC) (Kohli and Zhang 2013; Ito et al. 2011). Upon the ectopic expression of OKSM in somatic cells, both *Tet1* and *Tet2* have been shown to be upregulated during pluripotent reprogramming (Doege et al. 2012; Costa et al. 2013; Zhu et al. 2014; Sardina et al. 2018). The inhibition of *Tet1* and *Tet2* remarkably impaired the efficiency of the induction of iPSCs (Doege et al. 2012; Costa et al. 2013; Zhu et al. 2014; Sardina et al. 2018). TET1/TET2 physically interact with SOX2, NANOG, KLF4 or PARP1, which enable the positioning of TET1/TET2 to the loci of key pluripotency-associated genes by SOX2/NANOG/KLF4/PARP1 to initiate demethylation, increase chromatin accessibility and activate transcription (Doege et al. 2012; Costa et al. 2013; Zhu et al. 2014; Sardina et al. 2018). The 5fC/5caC demethylation pathway was lately demonstrated to be a major driver of epigenetic changes during pluripotent reprogramming, suggesting the regulation of epigenetic identity and cell fate changes by 5fC/5caC pathway (Caldwell et al. 2021). Taken together, the DNA methylation/demethylation machinery creates a permissive epigenetic profile, facilitating the completion of the pluripotent reprogramming.

#### The role of biomolecular condensates in pluripotent reprogramming

Emerging evidence supports the notion that biomolecular condensates are formed via liquid–liquid phase separation, which has been increasingly implicated in the compartmentalization and spatiotemporal coordination of biochemical reactions within cells (Mitrea et al. 2022; Mehta and Zhang 2022). Hnisz and colleagues proposed that phase-separated biomolecular condensates are the driving force of transcriptional control (Hnisz et al. 2017). The link between phase separation and transcriptional control is corroborated by the demonstration of phase separation of coactivator proteins (MED1 and BRD4) at super-enhancers (Sabari et al. 2018). The same group later reported that the transcription activation domain (AD) of OCT4 could phase-separated condensates with the Mediator coactivator at super-enhancers in embryonic stem cells (ESCs) (Boija et al. 2018). The influence of phase separation on pluripotent reprogramming was investigated in a recent study (Wang et al. 2021d). The authors demonstrated that the regulation of topological-associated domains (TAD) reorganization was mediated by the phase separation ability of OCT4, which remarkably improved the efficiency of somatic reprogramming (Wang et al. 2021d). This study provides strong evidence that phase separation of OCT4 exerts promoting effects on cell reprogramming by regulating the organization of higher-order chromatin structure (Fan et al. 2023). More recently, Zhu and colleagues described the coordination of biophysical signals and phase separation of YAP protein for the robust induction of iPSCs (Zhu et al. 2024). Cell-reprogramming-inspired dynamically responsive hydrogel was exquisitely fabricated to sense and faithfully respond to the metabolic remodeling and extracellular acidification during reprogramming, leading to the spontaneous stiffening of hydrogel (Zhu et al. 2024). The stiffening of cell-reprogramming-inspired dynamically responsive hydrogel elicited the nuclear translocation of YAP protein and the formation of YAP biomolecular condensates, which enable robust generation of iPSCs from human and mouse somatic cells (Zhu et al. 2024).

#### The inherent heterogeneity of pluripotent reprogramming

Low efficiency and stochasticity are two of the key features of pluripotent reprogramming process, implying that there exist heterogeneous cell types during the reprogramming process (Takahashi and Yamanaka 2016; Chen 2020). Earlier mechanistic studies of pluripotent reprogramming using bulk analysis have been interfered by the noise from other cell types without an eventual iPSC fate. The emerging of single-cell sequencing and ATAC-seq is well positioned to bridge this gap, enabling the capture of critical events happening in the rare cell populations that eventually become iPSCs (Chen 2020). Schiebinger et al. applied an optimal transport-based framework to capture the trajectory structure by calculating optimal cell couplings of probability distributions (Schiebinger et al. 2019). By using scRNA-seq data from > 315,000 cells gathered at half-day intervals across 18 days of pluripotent reprogramming, the authors described a conceptual framework to reconstruct the trajectories of reprogramming and found that the pluripotent reprogramming process unleashed a much wider range of developmental programs and subprograms than previously characterized (Schiebinger et al. 2019). In another study, Guo and co-workers developed a Single-cell Orientation Tracing-based algorithm, enabling to capture gene co-expression modules representing particular functions and construct the cell fate continuum of pluripotent reprogramming (Guo et al. 2019). In addition, the authors proposed that pluripotent reprogramming of somatic cells towards iPSCs was in the form of a series of bifurcating decisions, which mainly included three bifurcation point: the mesenchymal-to-epithelial transition (MET) bifurcations; the keratinocyte-like-NR/R bifurcations; the *Oct4/Dppa5a* bifurcations (Guo et al. 2019). Moreover, the authors predicted IFN-γ antagonist as reprogramming-promoting factors, which was validated in functional experiments (Guo et al. 2019).

### The molecular mechanisms governing direct reprogramming

The mechanisms underlying direct reprogramming have been recently reviewed elsewhere (Wang et al. 2021b, 2024b; Xie et al. 2023a). Here, we mainly spotlight key advances that contribute to our understanding of the intrinsic mechanism behind direct reprogramming.

#### Transcription factors

Lineage-enriched transcription factors are the driving forces of direct reprogramming. For neuronal reprogramming, insights have been gained from an elegant study on analyzing the mechanism of direct induction of neurons from fibroblasts via the ectopic expression of ASCL1, BRN2, and MYT1L (Wapinski et al. 2013). The authors observed that exogenous Ascl1 could bind to neuronal lineage-specific targets in fibroblasts at the early stage of neuronal induction and facilitate the recruitment of BRN2 to their binding sites at later stage (Wapinski et al. 2013). Therefore, ASCL1 acts as an “on-target pioneer factor” during neuronal reprogramming (Wapinski et al. 2013; Wang et al. 2021b). This study also discovered a unique trivalent chromatin signature (including H3K4me1, H3K27ac, and H3K9me3) in the host cells, which was predictive of the permissiveness for Ascl1 occupancy between different cell types (Wapinski et al. 2013). As mentioned in “pluripotent reprogramming” part of this review, OCT4, SOX2 and KLF4 belong to “off-target pioneer factors”, the binding regions of which in fibroblasts are largely different from the regions in pluripotent stem cells. Gata4 and MEF2C in cardiac reprogramming and FOXA3 in hepatic reprogramming are also pioneer factors (Wang et al. 2021c). Of interest, the pro-neural factor ASCL1 was demonstrated to possess cross-lineage potential (Wang et al. 2022a). The combination of ASCL1 and MEF2C driven the efficient reprogramming of fibroblasts into a mature cardiomyocyte-like cells (Wang et al. 2022a). Most cardiac reprogramming methods deployed a cocktail of transcription factors comprising of GATA4, MEF2C, and TBX5 to drive the cardiac reprogramming process. These three transcription factors were observed to exhibit combinatorial binding correlated with opening of chromatin particularly at cardiac lineage-specific loci (Stone et al. 2019). The use of transcription factor combinations to induce direct reprogramming raises an important question: are reprogramming factors equally important for the outcome of lineage conversion? Results from the investigations of the mechanisms underlying cardiac reprogramming have provided the notion that the stoichiometry of cardiac reprogramming factors not only impacts the efficiency of cardiac reprogramming but also the quality of induced cardiomyocyte-like cells (Wang et al. 2015a, 2015b). The authors showed that the optimal combination of reprogramming factors, which contained relatively higher MEF2C and lower GATA4 and TBX5 expression, could induce efficient cardiac reprogramming towards cardiomyocyte-like cells with higher quality (Wang et al. 2015a, 2015b), emphasizing the importance of the stoichiometry of reprogramming factors for robust direct reprogramming. The emerging of single-cell sequencing techniques enables researchers to deeply interrogate the transcriptional dynamics and heterogeneity during direct reprogramming. In a pioneer work led by Dr. Li Qian, the authors used single-cell RNA sequencing to analyze global transcriptome changes, reconstruct the reprogramming trajectory and uncover intermediate cell populations during cardiac reprogramming (Liu et al. 2017). Surface markers that allow the enrichment of induced cardiomyocytes during reprogramming was identified (Liu et al. 2017). Notably, single cell RNA-seq, ChIP-seq, ATAC-seq, and machine learning were leveraged in another study to investigate the mechanisms by which transcription factors acted during cardiac reprogramming (Stone et al. 2019). The authors provided strong evidence that GATA4, MEF2C and TBX5 exhibited interdependency of their binding patterns at specific loci, resulting in context-specific regulation of cardiac reprogramming (Stone et al. 2019). For hepatic reprogramming mediated by HNF4α and FOXA family member (FOXA1, FOXA2 or FOXA3), Horisawa and colleagues observed that FOXA proteins and HNF4α targeted chromatin and activated transcription of hepatic genes in a sequential and synergistic manner, which was required for the successful conversion of MEFs towards hepatocyte-like cells (Horisawa et al. 2020). Foxa3 was unique due to the ability of transferring from the distal to proximal regions of the transcription start sites of target genes, binding RNA polymerase II, and co-traversing target genes (Horisawa et al. 2020).

#### Chromatin remodeling and histone modifications

The ectopic expression of reprogramming factors elicited changes of chromatin accessibility at thousands of distal regions from the transcription start sites during the direct reprogramming process, which occurred as early as 12 h after enforced expression of reprogramming factors in starting fibroblasts (Wapinski et al. 2017). Wapinski and colleagues found that chromatin remodeling correlated with robust activation of endogenous neuronal genes (Wapinski et al. 2017), suggesting the coordinated epigenomic switch and transcriptional activation during direct reprogramming. The correlation between the extent of epigenome remodeling and the outcome of neuronal reprogramming was recently investigated by using wildtype form and phosphorylation-resistant form of NGN2 (PmutNGN2, with stronger neurogenic activity) (Pereira et al. 2024). The authors revealed that the faster and more efficient neuronal conversion mediated by PmutNGN2 was accompanied by more extensive multiscale epigenetic remodeling, such as enhancer–gene interaction sites, chromatin rewiring and DNA demethylation (Pereira et al. 2024). The detailed molecular events underlying neuronal reprogramming was interrogated by Janas and colleagues in a recent study. They demonstrated that neuronal-lineage transcription factors recruited Tip60 to the chromatin sites of a subset of silent, lineage-restricted genes, which was critical to the successful conversion of somatic cells towards neurons (Janas et al. 2022). Mechanistically, Tip60 mediated the acetylation of histone variant H2A.Z, which facilitated neuronal reprogramming by establishing epigenetic competence for bivalent gene activation and neuronal induction (Janas et al. 2022).

For cardiac reprogramming induced by GATA4, MEF2C, and TBX5, an early and rapid decreasing of H3K27me3 mark and increasing of H3K4me3 mark at cardiac loci was observed (Liu et al. 2016), which was correlated with transcriptional activation of cardiac-lineage genes. In contrast, a progressive increasing of H3K27me3 mark and decreasing of H3K4me3 mark at fibroblast loci occurred at later stage of cardiac reprogramming, which was accompanied by transcriptional repression in fibroblast-signature genes (Liu et al. 2016). These observations indicate that an early and fast unlocking of the cardiac program and a progressive shutdown of fibroblast program. The dynamics of enhancer landscape during cardiac reprogramming was recently interrogated by Hashimoto and co-workers (Hashimoto et al. 2019). The authors revealed that HAND2 and AKT1 recruited other reprogramming factors to cardiac enhancer elements, where they work synergistically for enhancer activation (marked by H3K27ac) at the initial stage of cardiac reprogramming (Hashimoto et al. 2019). More recently, a robust cardiac reprogramming system was developed using GATA4, TBX5, and a transcription-hyperactive form of MEF2C (via fusion with the transcription activation domain of MYOD) (Kojima et al. 2023). Mechanistic study demonstrated that the transcription-hyperactive form of MEF2C enhanced the efficiency and quality of cardiac reprogramming through p300-mediated chromatin remodeling (Kojima et al. 2023). In a notable study, Wang and colleagues integrated single-cell ATAC-seq and single-cell RNA-seq to dissect the chromatin accessibility dynamics and transcription changes during cardiac reprogramming (Wang et al. 2022b). Smad3 was identified to be a dual-functional transcription factor in cardiac reprogramming, acting as an impediment in the early stage of reprogramming and a facilitator during the intermediate stage of reprogramming (Wang et al. 2022b). Furthermore, the authors demonstrated the rapid establishment of H3K27ac-marked active cardiac enhancers at the early stage of reprogramming and the global remodeling of cis-regulatory interactions of cardiac genes along the reprogramming trajectory (Wang et al. 2022b).

Heterochromatin is defined as a chromosomal domain harboring repressive H3K9me3 mark or H3K27me3 mark and related factors that physically condense the chromatin (Wang et al. 2021b). H3K9me3-marked heterochromatin can restrict the binding by transcription factors, thus impeding gene activation and changes in cell identity (Wang et al. 2021b). Becker and colleagues found that H3K9me3-marked chromatin was the most refractory to gene activation by hepatic-enriched reprogramming factors (Becker et al. 2017). siRNA-mediated knockdown of RBMX and RBMXL1, two of H3K9me3 heterochromatin-associated proteins, led to the expression of liver-specific genes at early stages of reprogramming (Becker et al. 2017). In addition to these well-known histone marks, other forms of histone modifications, such as H2AK119Ub (the ubiquitylation of histone H2A at lysine 119) or H2BK120ub (the ubiquitylation of histone H2B at lysine 120), have been implicated to exert influence on direct reprogramming. For example, the polycomb ring finger oncogene *Bmi1* was identified as an epigenetic barrier to cardiac reprogramming (Zhou et al. 2016). shRNA-mediated knockdown of *Bmi1* resulted in the complete removal of H2AK119ub at cardiogenic loci, the increased level of H3K4me3 at cardiogenic loci and the significant upregulation of cardiogenic factors, indicating the suppressive role of H2AK119ub marks in cardiac reprogramming (Zhou et al. 2016). miRNAs also have the capability to directly convert fibroblasts to a cardiomyocyte-like cells. A miRNA combo consisting of miR-1, miR-133a, miR-208a and miR-499 enabled the direct induction of cardiomyocyte-like cells from fibroblasts of different species (such as mice, pigs, dogs and humans) (Jayawardena et al. 2012; Baksh and Hodgkinson 2022). Mechanistic studies suggest that cardiac reprogramming induced by miR combo requires the demethylation of H3K27me3 at cardiogenic loci, which is achieved via concomitant modulation of the expression of H3K27 methyltransferase and demethylase (Dal-Pra et al. 2017).

#### DNA methylation

Direct reprogramming is also accompanied by extensive reconfiguration of DNA methylation. During direct reprogramming of fibroblasts towards cardiomyocyte-like cells, the promoters of *Myh6* and *Nppa*, two cardiac lineage genes, were demethylated as early as day 3 after GMT induction (Liu et al. 2016). DNA methylation dynamics has also been implicated in neuronal reprogramming. For example, Luo and colleagues demonstrated the dynamics of DNA methylation during neuronal reprogramming (Luo et al. 2019). The authors found that co-expression of Ascl1, Brn2 and Mytl1 in fibroblasts elicited the establishment of a global non-CG methylation (mCH) landscape, which is an epigenomic signature of mature cortical neurons (Luo et al. 2019). Ascl1 was observed to display pioneer functions in this regard by inducing local demethylation at most of its binding sites (Luo et al. 2019). Interestingly, the authors found that de novo DNA methylation was also required for efficient reprogramming, which was supported by the observation that ablation of Dnmt3a in fibroblasts resulted in reduced reprogramming potential (Luo et al. 2019). In a notable study, Song and co-workers investigated the influence of mechanical forces on the modulation of the epigenetic state and the outcomes of neuronal reprogramming (Song et al. 2022). The authors found that millisecond deformation of the nucleus of reprogramming cells led to the decrease of histone methylation (H3K9me3) and DNA methylation, which enhanced the conversion of fibroblasts into neurons (Song et al. 2022). Consistent with this observation, pretreatment of fibroblasts with decitabine, a DNA methyltransferase inhibitor, enhanced the efficiency of neuronal reprogramming (Song et al. 2022). More recently, Pereira and colleagues demonstrated the relationship of epigenome remodeling with the outcome of mouse astrocyte-to-neuron reprogramming using phosphorylation-resistant form of Ngn2 (PmutNgn2), which exhibited stronger neurogenic activity compared with the wildtype form of Ngn2 (Pereira et al. 2024). The authors found that the improved astrocyte-to-neuron reprogramming mediated by PmutNgn2 was accompanied by more extensive epigenetic changes, including the higher degree of DNA demethylation (Pereira et al. 2024).

## Applications of cell reprogramming

The fast evolving of cell reprogramming technology has significantly transformed the landscape in biomedical research and provided powerful approaches to derive functional cell types of interest. Cell reprogramming techniques have been broadly used in developmental biology, disease modeling, drug development, cell therapies, and cancer immunotherapy. The utilization of cell reprogramming for in vitro modeling of human embryonic development has recently been reviewed elsewhere (Cerneckis et al. 2024; Yagi et al. 2024) and will therefore not be discussed in detail here. Here, we focus on the key advances in the widespread applications of cell reprogramming for disease modeling, drug development, cell therapies, and cancer immunotherapy.

### Disease modeling

Prior to the advent of iPSC-derived cells/organoid technology, researchers typically capitalize on animal models for disease modeling (Kim et al. 2020; Rowe and Daley 2019). The generation of animal models for a given disease requires prior knowledge of the causative conditions or genes involved. Even with the fast evolving of CRISPR/Cas9-mediated gene editing technology, it usually takes more than a year to generate genetically modified animal models that mimic human diseases (Kim et al. 2020). In addition, the construction of animal models represents a highly technical threshold and requires professionals who are proficient in embryo manipulation techniques. Furthermore, species-specific differences between animal models and humans, such as the microbiota and pathogen composition or infection dynamics in lung and brain, limit the translation to humans of certain phenomena observed in animal models, restraining the utility of animal models to authentically simulate human diseases (Kim et al. 2020). The gaps between animal models and humans are expected to be bridged through iPSC-derived cells/organoids, which are relatively easier to handle and can be established within a few weeks or months (Rowe and Daley 2019).

The iPSC technology used to mimic human diseases is characterized by the fact that iPSCs can be reprogrammed from somatic cells of patients with specific diseases and carrying pathogenic mutations or genetic risk factors (Park et al. 2008a). In most cases, iPSCs with disease-associated genetic backgrounds undergo stepwise differentiation into cell types of interests that can faithfully reconstruct disease-specific phenotypes (Park et al. 2008a; Rowe and Daley 2019), which help to deepen the understanding of disease mechanisms and develop effective therapies. Disease phenotypes at cellular level have been recapitulated in cells differentiated from patient-specific iPSCs across a number of diseases, such as Alzheimer’s disease (Israel et al. 2012), Parkinson’s disease (Bose et al. 2022; Kim et al. 2024), long-QT syndrome (Moretti et al. 2010; Wuriyanghai et al. 2018), schizophrenia (Brennand et al. 2011; Toritsuka et al. 2021), dysautonomia (Lee et al. 2009, 2012), spinal muscular atrophy (Ebert et al. 2009; Ohuchi et al. 2016), Fanconi anaemia (Liu et al. 2014; Marion et al. 2020), or infectious diseases (Ye et al. 2014; Lafaille et al. 2012). These endeavors have remarkably deepened our understanding of the mechanisms underlying modelled disorders.

For many diseases, precise modeling of cell–cell interactions within 3D structures contributes to recapitulating important aspects of disease pathophysiology. Co-culture systems represent one of the methods used for the generation of more complex disease models. In a notable study, Giacomelli and colleagues described a feasible and reproducible 3D microtissue system composed of cardiomyocytes, cardiac endothelial cells, and cardiac fibroblasts, derived entirely from iPSCs (Giacomelli et al. 2020). This tri-culture approach led to the improved sarcomere structure and key structural features of more mature cardiomyocytes (Giacomelli et al. 2020). Then, arrhythmogenic cardiomyopathy (ACM) was modeled using ACM patient-specific iPSCs carrying a PKP2 premature stop mutation (Giacomelli et al. 2020). ACM patient iPSC-derived cardiac fibroblasts (ACM-CFs), as the only diseased cellular component, were incorporated into the 3D microtissue system (Giacomelli et al. 2020). The authors found that ACM-CFs induced arrhythmic behavior in wild-type cardiomyocytes. Interestingly, ACM-CFs were characterized by the reduced expression of CX43 and the higher propensity to assume a myofibroblast-like identity, which may both impact electrical conduction and increase the risk of arrhythmia (Giacomelli et al. 2020).

The rapid advances in organoid technology have made it possible to develop complex iPSC-derived organoids to more faithfully reproduce human disease pathophysiology at tissue level or organ level. iPSC-derived organoids have been leveraged to remodel a broad spectrum of diseases, such as Alzheimer’s disease (Fernandes et al. 2024; Zhao et al. 2020), Parkinson's disease (Kim et al. 2023), Huntington's disease (Conforti et al. 2018), Lewy body disease (Jin et al. 2024), microcephaly (Sun et al. 2020; Lancaster et al. 2013), Down syndrome (Tang et al. 2021), autism spectrum disorder (de Jong et al. 2021), Miller–Dieker syndrome (Bershteyn et al. 2017), steatohepatitis (Ouchi et al. 2019), cholestasis (Shinozawa et al. 2021), retinitis pigmentosa (Lane et al. 2020), infectious diseases (Nie et al. 2018; Zhou et al. 2017b; Yang et al. 2020; Tiwari et al. 2021; McMahon et al. 2021; Han et al. 2022) or rare diseases (Deng et al. 2023; Ogawa et al. 2015). To date, animal studies of Alzheimer’s disease (AD) have mostly focused on genetic mutations linked to inherited, early-onset autosomal dominant Alzheimer’s disease (ADAD). It remains a significant challenge to mimic the age-associated neuropathological hallmarks of sporadic, late-onset AD (LOAD), accounting for the vast majority of cases. iPSCs–derived cortical neurons/brain organoids have limited ability to reflect age-associated characteristics due to the finding that the induction of iPSCs from aged donor cells is accompanied by resetting ageing-related traits of donor cells (Lapasset et al. 2011; Miller et al. 2013). Previous studies have established the notion that direct neuronal reprogramming retains age-associated profiles pre-existed in the original donor cells, such as transcriptional landscape, epigenetic signatures, and telomere length (Huh et al. 2016; Mertens et al. 2015). Modeling late-onset Alzheimer’s disease neuropathology was described in a recent study led by Yoo (Sun et al. 2024). In this study, the authors demonstrated microRNA (miRNA)-mediated, 3D-direct neuronal reprogramming from fibroblasts of LOAD patients, ADAD patients or age-matched normal individuals (Sun et al. 2024). In comparison with inherited autosomal dominant Alzheimer’s disease, LOAD has distinct molecular features, among which retro-transposable elements were closely associated with the progression of LOAD (Sun et al. 2024). Furthermore, the authors found that inhibition of retro-transposable elements with lamivudine had a positive influence on the phenotypes of LOAD (Sun et al. 2024). To sum, this elegant study provides a conceptual framework to model the authentic, age-associated neuropathology of LOAD and make therapeutic interventions.

### Drug development

Previous studies have established the notion that patient’s genetic background can influence drug response and even trigger unnecessary side effects (Rowe and Daley 2019), patient-specific iPSC-derived cells/organoids offer significant advantages in drug development applications. For example, a simplified two-step neuronal differentiation protocol was established using an iPSC line that stably expressed doxycycline-inducible mouse Ngn2 transgene (Wang et al. 2017a). The authors developed a robust high-content screen system to identify tau-lowering compounds using iPSC-derived glutamatergic neurons and discovered adrenergic receptor agonists to decrease the level of endogenous tau (Wang et al. 2017a). In another study, Brownjohn and colleagues described a feasible strategy to perform phenotypic, small-molecule screening in iPSC-derived Alzheimer’s disease model (Brownjohn et al. 2017). Avermectins were identified as γ-secretase-independent modulators of amyloid precursor protein processing (Brownjohn et al. 2017). Gutbier and co-workers described an approach for the large-scale production of human iPSC-derived macrophage, which were utilized to identity compounds that modulate phagocytosis, cytokine release, calcium release and chemotaxis (Gutbier et al. 2020). Recently, Theodoris and colleagues demonstrated a conceptual framework to perform network-based drug screening in a heart disease model based on iPSC-derived endothelial cells (Theodoris et al. 2021). With the help of machine learning, the authors were able to identify XCT790 that reversed the gene network dysregulated by *Notch1* haploinsufficiency in in calcific aortic disease (Theodoris et al. 2021). More recently, key pathological features of Lewy body disease (LBD) were faithfully recapitulated in cortical organoids derived from LBD patient-specific iPSCs carrying *α-synuclein* (*SNCA*) gene triplication (Jin et al. 2024). Through functional screening, the authors identified four Food and Drug Administration–approved drugs (entacapone, tolcapone, phenazopyridine, hydrochloride, and zalcitabine) that targeted α-SYN aggregation and restored mitochondrial dysfunction in iPSC-derived LBD model (Jin et al. 2024).

iPSC-derived cells/organoids have also been applied to the scenarios of drug pharmacokinetic, pharmacodynamic and toxic testing. For example, pharmacokinetic evaluation systems have been established with human iPSC-derived functional intestinal organoids, which not only expressed genes encoding drug transporters but also genes encoding efflux transporters or drug-metabolizing enzyme CYP3A4 (Onozato et al. 2018; Kwon et al. 2021). A novel strategy for in vivo pharmacodynamic evaluations has been recently demonstrated by transplantation of iPSC-derived kidney organoids into athymic rats, enabling the studies of an investigational new drug, GFB-887 (Westerling-Bui et al. 2022). Notably, cardiac organoids have been successfully derived from iPSCs, allowing for the recapitulation of 3D tissue-level responses to drug-induced/exacerbated cardiotoxicity (Richards et al. 2020). In another study, Sharma and co-workers utilized human iPSCs–derived cardiomyocytes, endothelial cells or cardiac fibroblasts to systematically evaluate cell type–specific cardiotoxicities of tyrosine kinase inhibitors (Sharma et al. 2017). Complex multicellular kidney organoids with fully segmented nephrons have also been derived from human iPSCs to investigate the nephrotoxicity of cisplatin (Takasato et al. 2015).

Recently, different types of organoids-on-chips have been developed for the systematic studies of drug pharmacokinetics and pharmacodynamics. Organoids-on-chips mean the production of in vitro organotypic models with more physiological relevance, which is achieved through the convergence of organoids and organs-on-a-chip technology (Park et al. 2019; Wang and Qin 2023). Organs-on-a-chip is a microfabricated cell culture device aiming to model key architectural and functional features of human organs by precise control of fluid flow, biochemical factors, mechanical conditions, cell–cell/cell–matrix interactions and tissue–tissue/organ–organ interactions (Park et al. 2019; Wang and Qin 2023). Organoids-on-a-Chip has significantly contributed to the holistic investigations of the transportation, absorption, distribution, metabolism, and excretion of drugs (Osaki et al. 2018; Sakolish et al. 2021; Park et al. 2020; Wang et al. 2018; Schneider et al. 2019; Abulaiti et al. 2020; Fanizza et al. 2022; Huebsch et al. 2022), which may revolutionize the pipeline of drugs development. Comparing to the existing drug screening platform, organoids-on-chips can not only offer unique insights into the transportation, absorption, distribution, metabolism, and excretion of drugs in more physio- and pathological scenarios, but also be beneficial for the dynamic monitoring of the complex interactions between drugs and multi-organs.

### Cell therapy

The increasingly translational success of cell therapy into clinical applications has raised expectations that cell therapy will heal injured, ageing, and even some refractory diseases in the near future (Yamanaka 2020; Zhu et al. 2023b) (Table 2). A typical example is the successful treatment of hematological malignancies, such as acute lymphoblastic leukemia and large B cell lymphoma, using chimeric antigen receptor (CAR) T cells (Bashor et al. 2022). However, the limited proliferation capability and unwanted heterogeneity of primary T cells or natural killer (NK) cells significantly inhibit their widespread applications (Cerneckis et al. 2024). iPSC technology can be deployed to circumvent these dilemmas, as iPSCs are amenable to be engineered, unlimitedly self-replicated, and differentiated into almost all cell types of interests. iPSCs-based cell therapies have been used to treat a spectrum of diseases, such as age-related macular degeneration (Mandai et al. 2017), neurodegenerative disease (Kikuchi et al. 2017; Song et al. 2020a; Doi et al. 2020; Tao et al. 2021; Hiller et al. 2022; Brot et al. 2022), liver disease (Choi et al. 2020; Pouyanfard et al. 2021), heart disease (Lancaster et al. 2019; Yeung et al. 2019; Jiang et al. 2020), diabetes (Maxwell et al. 2020; Balboa et al. 2022; Du et al. 2022; Wang et al. 2024c), or limbal stem-cell deficiency (LSCD) (Soma et al. 2024).

**Table 2:** Representative stem cell-based regenerative therapies approved or in clinical trials for treating damaged, aging or intractable diseases

Cell resources	Conditions or diseases	Clinical trial ID	First posted	Clinical stage
iPSC-derived retinal pigmented epithelial cells	Age-related macular degeneration	NCT02464956	June 8, 2015	Completed
iPSC-derived retinal pigmented epithelial cell sheet	Age-related macular degeneration	UMIN000011929	October 2, 2013	Completed
iPSC-derived retinal pigmented epithelial cells	Age-related macular degeneration	NCT04339764	April 9, 2020	Recruiting
iPSC-derived engineered heart myocardium	Heart failure	NCT04396899	May 21, 2020	Recruiting
Allogenic human pluripotent stem cell-derived cardiomyocytes	Heart failure	NCT03763136	December 4, 2018	Recruiting
iPSC-derived cancer antigen-specific T cells	Gastrointestinal cancers Breast cancers Pancreatic cancers Melanoma Lung cancers	NCT03407040	January 23, 2018	Terminated
Induced pluripotent stem cells	Imprinted disorders	NCT05214742	January 19, 2022	Enrolling by invitation
iPSCs carrying monoamine transporter polymorphisms	Drug addiction	NCT01534624	February 7, 2012	Completed
Induced pluripotent stem cells	Autism spectrum disorders	NCT02720939	January 1, 2016	Completed
Human induced pluripotent stem cells	Diabetic Retinopathy	NCT03403699	January 11, 2018	Recruiting
Human iPSC-derived cardiomyocytes	Heritable cardiac arrhythmias	NCT02413450	August, 2013	Enrolling by invitation
Human iPSC-derived cardiomyocytes	Congenital heart diseases	NCT05647213	February 3, 2023	Recruiting
Human iPSC-derived β cells encapsulated within bioengineered device	Type 1 diabetes mellitus	NCT02239354	September 12, 2014	Terminated
Human iPSC-derived β cells	Type 1 diabetes mellitus	ChiCTR2300072200	June 6, 2023	Recruiting
Human iPSC-derived corneal epithelium	Limbal stem-cell deficiency symptom	UMIN000036539	May 23, 2019	Completed
Human iPSC-derived retinal cells	Age-related macular degeneration	NCT05991986	August, 2023	Not yet recruiting
Human iPSC-derived brain organoids	High grade astrocytoma	NCT03971812	June 7, 2019	Unknown status
Human iPSC-derived optic vesicle organoids	Ocular developmental disease	NCT06408701	May 2, 2024	Not yet recruiting

Recent years have also witnessed the therapeutic interventions of numerous types of diseases using cell therapies based on direct reprogramming technology. For example, functional repair of damaged brains after ischemic injury (Chen et al. 2020), spinal cord injury (Puls et al. 2020; Talifu et al. 2024), optic nerve crush injury (Wang et al. 2024a), and glaucoma (Soucy et al. 2023), using neuronal reprogramming-based in situ endogenous regeneration, has been documented. In a proof-of-concept study, Qian and colleagues reported the direct conversion of resident cardiac fibroblasts in the murine heart into cardiomyocyte-like cells in vivo by retroviral delivery of Gata4, Mef2c and Tbx5 (GMT), demonstrating the feasibility of using in vivo cardiac reprogramming for heart repair (Qian et al. 2012). In a notable study led by Ieda, the authors described the delivery of Gata4, Mef2c and Tbx5 mediated by the non-integrating Sendai virus, which was safer and more efficient than retroviral-GMT in reprogramming resident cardiac fibroblasts into cardiomyocyte-like cells in mouse infarct hearts (Miyamoto et al. 2018). The recovery of cardiac function and the reduction of fibrosis after myocardial infarction was observed after GMT delivery. In vivo hepatic reprogramming-based treatment of liver diseases has also been developed (Huang et al. 2011; Jiang et al. 2023). For example, Rezvani and colleagues demonstrated a therapeutic strategy for the treatment of liver fibrosis using adeno-associated virus (AAV)-delivered hepatic transcription factors, which mediated the conversion of resident myofibroblasts into hepatocyte-like cells in mice, replicating the proliferative capacity of functional hepatocytes and reducing liver fibrosis (Rezvani et al. 2016). The only effective treatment for end-stage liver diseases is liver transplantation, which has been dampened due to the shortage of donor livers. Alternative therapies, such as hepatocyte transplantation, have been recently developed for advanced liver diseases. To tackle the limited proliferation capacity of functional hepatocytes, Hishida and co-workers described the direct reprogramming of hepatocytes into hepatic progenitor cells with increased proliferative capacity, which enabled the regeneration of injured liver in a mouse model (Hishida et al. 2022).

Previous studies have demonstrated that the induction of iPSCs from aged donor cells is accompanied by resetting ageing-related characteristics of donor cells (Lapasset et al. 2011; Miller et al. 2013), which laid foundations for the recent discoveries of partial reprogramming-induced rejuvenation (Ocampo et al. 2016; Simpson et al. 2021; Sarkar et al. 2020; Chondronasiou et al. 2022; Gill et al. 2022; Olova et al. 2019). Partial reprogramming has been recently documented to restore vision in aged mice (Lu et al. 2020) or to extend the life span of mice of premature aging or physiological aging (Ocampo et al. 2016; Browder et al. 2022), demonstrating the therapeutic potential of partial reprogramming. In a notable study led by Belmonte, the authors demonstrated a targeted partial reprogramming approach using adeno-associated viruses to deliver *OSK* under the control of the *cyclin-dependent kinase inhibitor 2a* (*Cdkn2a*) promoter, which allowing for specific and partial reprogramming of aged and stressed cells in a mouse model of Hutchinson-Gilford progeria syndrome (HGPS) (Sahu et al. 2024). Treated mice exhibited enhanced resilience, improved overall fitness, and extended life spans. Taken together, these studies indicate that partial reprogramming represents a powerful approach to treat damaged or ageing diseases (Sahu et al. 2024). In addition to the therapeutic potential for Hutchinson-Gilford progeria syndrome (HGPS), the partial reprogramming strategy has been utilized to develop potential therapy for myocardial infarction. For example, Chen and colleagues demonstrated the therapeutic potential of partial reprogramming for myocardial infarction (Chen et al. 2021). The authors found that short-term, cardiomyocyte-specific expression of Oct4, Sox2, Klf4 and c-Myc (OSKM) before and during myocardial infarction resulted in the amelioration of myocardial damage and the improved cardiac function (Chen et al. 2021). Taken together, these studies demonstrate that partial reprogramming holds great promises for the treatment of diversified types of diseases.

### Cancer immunotherapy

The concept and strategy of cell reprogramming technology have recently been leveraged to develop cancer immunotherapy. In a proof-of-concept study led by Pereira, the authors reported the successful reprogramming of 36 cancer cell lines derived from human/mouse hematological and solid tumors into professional antigen-presenting cells (tumor cell-derived APCs) via the enforced expression of the transcription factors PU.1, IRF8, and BATF3 (Zimmermannova et al. 2023). Tumor-derived APCs exhibited the expression of antigen presentation complexes and costimulatory molecules on their surface, enabling the presentation of endogenous tumor antigens on MHC-I and promoting killing by CD8 + T cells (Zimmermannova et al. 2023). Recently, the same group demonstrated the direct reprogramming of cancer cells into immunogenic conventional type I dendritic cell-like cells (tumor cell-derived cDC1) in vivo by adenoviral-mediated delivery of the transcription factors PU.1, IRF8, and BATF3, resulting in the reconstruction of the tumor microenvironment, the formation of tertiary lymphoid structures, and the establishment of systemic immunity (Ascic et al. 2024). More recently, we have developed iPSC membrane-derived prophylactic cancer vaccine, which inhibited tumor progression by eliciting systemic anti-tumor memory T-cell and B-cell immune responses in several preclinical tumor models, such as melanoma, colon cancer, breast cancer and post-operative lung metastasis (Li et al. 2024b). In addition, iPSC membrane-derived prophylactic tumor vaccine was observed to have favorable safety profile (Li et al. 2024b). To sum, the authors described a novel strategy for the design of a universal cancer prevention vaccine harnessing surface antigens shared by tumor cell-derived membrane and iPSC-derived membrane.

## Conclusions and perspectives

**Fig. 4:**
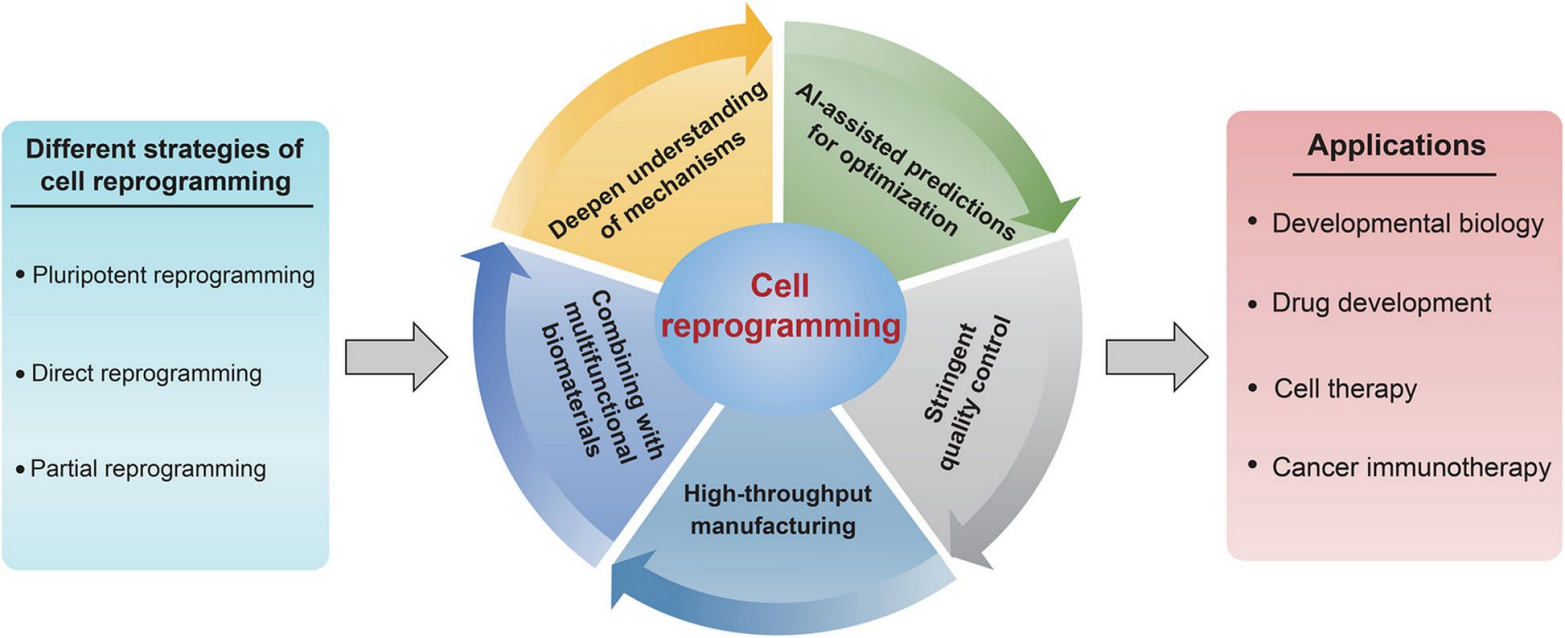
Future directions for cell reprogramming-based cell therapies. AI: artificial intelligence. Cell reprogramming-based techniques have been broadly used in developmental biology, disease modeling, drug development, cell therapies, and cancer immunotherapy. For example, ectopic expression of defined reprogramming factors in fibroblasts reportedly give rise to both embryonic-like cell types (iPSCs) and extra-embryonic-like cell types (trophoblast stem cells), which can self-assemble into 3D blastocyst-like models, providing unique insights into early embryonic development. For disease modeling, iPSC-derived cells/organoids have been used to faithfully recapitulate key pathological features of diversified diseases, such as neurological diseases (Alzheimer’s disease, Parkinson’s disease, microcephaly), metabolic diseases (steatohepatitis, cholestasis), infectious disease (COVID-19) or rare diseases. Modeling late-onset Alzheimer’s disease (LOAD) neuropathology was described in a recent study, which derived cortical neurons from fibroblasts of LOAD patients using microRNA-mediated, 3D-direct neuronal reprogramming. For drug development, iPSC-derived cells/organoids have been increasingly applied to the scenarios of drug pharmacokinetic, pharmacodynamic and toxic testing. In addition, the integration of iPSC-derived organoids with organ-on-chips will enable systemic investigations of drug pharmacokinetics and pharmacodynamics. The increasing success of iPSC-derived cells/organoids in clinical trials has raised expectations that iPSCs-based cell therapy will heal injured, ageing, and refractory diseases in the near future. The combination of iPSCs-based cell therapy with multifunctional biomaterials will address the post-transplantation issues of cell survival, retention, and engraftment into the host tissues. In recent years, the strategy of partial reprogramming has been utilized to develop therapies for ageing disease or myocardial infarction. The advancement of novel partial reprogramming technology will hold great promises for treating more types of diseases in the future. Last but not least, the concept and strategy of cell reprogramming technology have been leveraged to develop cancer immunotherapy, such as direct reprogramming of cancer cells towards professional antigen-presenting cells, resulting in the reconstruction of the tumor microenvironment and the establishment of systemic immunity against tumors. Direct reprogramming of cancer cells towards other types of immune cells, such as macrophage or NK cells, will empower the host’s immune responses against tumors. Moreover, an iPSC membrane-derived prophylactic cancer vaccine has recently been developed (Li et al. 2024b), which inhibited tumor progression by eliciting systemic anti-tumor memory T-cell and B-cell immune responses in several preclinical tumor models. The advancement of cell reprogramming technology will undoubtedly provide opportunities to develop novel immunotherapies against diversified types of tumors

Since the initial report of fibroblast-to-myoblast fate reprogramming in 1987, the past four decades have witnessed the burgeoning development of methodologies for cell reprogramming, the in-depth dissections of mechanisms governing cell reprogramming, and the widespread applications of cell reprogramming in developmental biology, disease modeling, drug development, cell therapy, and cancer immunotherapy. Positive data from two recent clinical trials has strengthened our confidence that cell reprogramming-based therapies will treat a wide range of diseases in the near future (Soma et al. 2024; Wang et al. 2024c). Despite these advances, there are several challenges that need to be faced and addressed, such as how to better understand the mechanisms underlying cell reprogramming with state-of-the-art single cell omics and spatial transcriptome, how to utilize artificial intelligence/computational models to predict critical conditions and reprogramming cocktails for precise and robust cell reprogramming, how to establish and constantly revise the standards and specifications of critical quality attributes (CQAs, such as cell identity, genetic stability, viability, purity and potency, microbial safety, biosafety, and infectious pathogens) for quality control of cells/organoids (Sullivan et al. 2018; Wang et al. 2024c), how to integrate high-throughput manufacturing platforms to produce sufficient numbers of clinical-grade functional cells/organoids for cell therapy (Fig. 4). One of critically important aspects in monitoring the manufacture of cell/organoid products is quality control under the policy of Good Manufacturing Practice (Sullivan et al. 2018; Hao et al. 2020b, 2020a; Zhang et al. 2022), which includes but not limits to stem cell assembly, handling, storage, personnel training, and equipment of the cell processing laboratory.

There are several major challenges such as tumorigenicity, immunogenicity, the post-transplantation issues of poor cell survival, retention, and engraftment into host tissues, which need to be addressed so as to maximize the potential of iPSC-based cell therapy (Yamanaka 2020; Zhu et al. 2023b). The tumorigenic concern of iPSC-based cell therapy may be caused by the existence of undifferentiated and/or immature cells in the final iPSC-derived cell products; the persistent activity of tumorigenic reprogramming factors in iPSCs; genetic mutations that have occurred during in vitro culture of iPSCs (Yamanaka 2020). These challenges have been progressively addressed through the establishment of robust methods of in vitro directed differentiation of iPSCs into desired cell types (Mandai et al. 2017; Du et al. 2022; Wu et al. 2023; Lee et al. 2024); the development of stringent purification procedures to purify the desired cells and eliminate the undesired/undifferentiated cells (Hayashi et al. 2017, 2018; Sougawa et al. 2018); the more controllable and safer generation of high-quality iPSCs via small chemicals but not cancer-causing transgenes (Hou et al. 2013; Guan et al. 2022; Liuyang et al. 2023); the circumvention of immune responses via the utilization of autologous iPSC-derived cell products (Mandai et al. 2017; Sharma et al. 2019; Wang et al. 2024c) or HLA-matched iPSC-derived cell products (Sugita et al. 2016, 2020; Kawamura et al. 2016; Xu et al. 2019). In addition, functional bioengineered materials have been fabricated to address the post-transplantation issues of poor cell survival (Führmann et al. 2016; Mitrousis et al. 2018; Lee et al. 2018; Moshayedi et al. 2016), retention (Ballios et al. 2015; Wang et al. 2017b), and engraftment into host tissues (Jha et al. 2015; Tigner et al. 2024). Therefore, the integration of iPSC-based cell therapy with multifunctional biomaterials will undoubtedly improve the therapeutic outcomes of iPSCs/iPSC-derived cell products.

Standards are expected to be established to form large-scale, commercial systems for the broad applications of cell reprogramming-based therapies (Fig. 4). Also, accessibility and affordability should be taken into account when translating cell reprogramming-based therapies into clinical scenarios. Finally, the integration of multifunctional biomaterials into cell reprogramming-based therapies is predictable to improve the therapeutic outcomes and meet the unmet needs to treat injured, degenerative, aging, and other intractable diseases.

## Data Availability

Not applicable.

## Abbreviations

MEFs: Mouse embryonic fibroblasts
TTFs: Tail-tip fibroblasts
MDFs: Mouse dermal fibroblasts
HEFs: Human embryonic fibroblasts
HDFs: Human adult fibroblasts
HFFs: Human fetal limb fibroblasts
HPFs: Human pulmonary fibroblasts
IMR90: Human embryonic lung fibroblasts
hADSCs: Human adult adipose-derived mesenchymal stromal cells
hASFs: Human adult skin dermal fibroblasts
PBMCs: Peripheral blood mononuclear cells
ESCs: Embryonic stem cells
iPSCs: Induced pluripotent stem cells
CiPS: Chemically induced pluripotent stem cells
hCiPS: Human chemically induced pluripotent stem cells
YAP: Yes-associated protein
CHIR99021: GSK-3 signaling inhibitor
DAPT: Notch pathway inhibitor
Forskolin: Activates adenylyl cyclase and increases the intracellular levels of cAMP
I-BET151: Inhibitors of BET family proteins
ISX9: Activator of Ca2 + signaling
Y-27632: ROCK inhibitor
LDN193189: BMP signaling inhibitor
SAG: Sonic hedgehog agonist
SB431542: TGFβ inhibitor
CRISPR: Clustered regularly interspaced short palindromic repeats
MET: Mesenchymal-to-epithelial transition
CAR-T: Chimeric antigen receptor (CAR) T cells
LSCD: Limbal stem-cell deficiency
HGPS: Hutchinson-Gilford progeria syndrome
CQAs: Critical quality attributes
AI: Artificial intelligence
